# Global burden of lung cancer in women of childbearing age attributable to ambient particulate matter pollution: 1990–2021

**DOI:** 10.1002/cam4.70241

**Published:** 2024-09-24

**Authors:** Ying‐da Song, Ruizhe Wang, Jia‐xuan Wang, Xun‐wu Tan, Jun Ma

**Affiliations:** ^1^ Department of Thoracic Surgery Shanxi Provincial People's Hospital Taiyuan Shanxi China; ^2^ Fifth Clinical Medical College, Shanxi Medical University Taiyuan Shanxi China; ^3^ First Clinical Medical College, Changzhi Medical College Changzhi Shanxi China; ^4^ Second Clinical Medical College, Changzhi Medical College Changzhi Shanxi China

**Keywords:** ambient particulate matter air pollution, epidemiology, Global Burden of Disease 2021, lung cancer, women of childbearing age

## Abstract

**Background:**

This study aimed to evaluate the global burden of lung cancer due to ambient particulate matter (PM) pollution in women of childbearing age from 1990 to 2021.

**Methods:**

This was a secondary analysis utilizing data from the Global Burden of Disease (GBD) 2021, with a focus on the temporal trends of the lung cancer burden attributable to ambient PM2.5 among women of childbearing age.

**Results:**

In 2021, the global mortality and disability‐adjusted life years (DALYs) number of lung cancer burden attributable to ambient PM2.5 among women of childbearing age were approximately 5205 and 247,211, respectively. The rate of lung cancer attributable to ambient PM2.5 among women of childbearing age increased between 1990 and 2021, with the age‐standardized mortality rate (ASMR) increasing from 0.22 (95% uncertainty interval [UI]; 0.13 to 0.33) to 0.25 (95% UI; 0.14 to 0.37; average annual percent change [AAPC] = 0.40) and the age‐standardized DALYs rate (ASDR) increasing from 10.39 (95% UI; 5.96 to 15.72) to 12.06 (95% UI; 6.83 to 17.51; AAPC = 0.41). The middle sociodemographic index (SDI) region, East Asia, and China had the heaviest burden, while the high SDI region showed the highest decrease. ASMR and ASDR exhibited an inverted U‐shaped relationship with the SDI.

**Conclusions:**

From 1990 to 2021, the lung cancer burden attributable to ambient PM2.5 among women of childbearing age exhibited an increasing trend. Furthermore, increasing attention should be paid to the middle SDI region, East Asia, and China, as ambient PM pollution remains a critical target for intervention.

## 1 INTRODUCTION

Cancer is a significant global public health issue, with lung cancer having the highest incidence and mortality rates. According to the *GLOBOCAN* 2022 database, there was 2.5 million new cases in 2022, accounting for 12.4% of all new cancer cases. Globally, 1.8 million people succumb to lung cancer, constituting 18.7% of all cancer‐related deaths.^1^ Exposure to risk factors for lung cancer has broadly changed with socioeconomic development and time. In addition, air pollution has emerged as a major driver of these environmental risk factors.^2^, ^3^

Air pollution is a major global health issue and is ranked as the second largest risk factor for cancer worldwide.^4^, ^5^, ^6^ The primary contributor to the global air pollution burden is particulate matter (PM) pollution, which accounts for 8.0% of all disability‐adjusted life years (DALYs). PM pollution primarily encompasses household PM air pollution and ambient PM air pollution.^7^ From 2000 to 2021, the attributable DALYs due to global household air pollution significantly declined, while those attributable to ambient PM pollution and ambient ozone pollution increased.^5^

Recent studies have emphasized that in addition to traditional risk factors, such as smoking, ambient PM pollution significantly contributes to the development of lung cancer.^8^, ^9^ Fine particulate matter (PM2.5) within ambient PM pollution has been identified as a significant risk factor for lung cancer.^10^ In recent years, research has indicated an increasing incidence of lung cancer among nonsmoking women, particularly young women.^11^, ^12^, ^13^ Furthermore, ambient PM2.5 was ranked as the second largest risk factor for female lung cancer DALYs and mortality across all ages in 2021, while it emerged as a primary risk factor for lung cancer in women of childbearing age.^5^ Women of childbearing age constitute a particularly vulnerable demographic group due to their unique physiological and social conditions, which include hormonal fluctuations, reproductive health considerations, and potential pregnancy‐related complications contribute to the complexity of their health conditions. Despite growing awareness of the impact of ambient PM pollution on lung cancer, comprehensive analyses focusing on this demographic remain rare. Currently, the epidemiology of the lung cancer burden due to ambient PM2.5 among women of childbearing age remains unknown at the global, regional, and national levels.

The Global Burden of Disease (GBD) study offers new perspectives for understanding disease distribution, trends, and risk factors.^14^ Therefore, this study aimed to determine the trends of lung cancer epidemiology due to ambient PM2.5 among women of childbearing age by analyzing age‐standardized mortality rate (ASMR) and age‐standardized disability‐adjusted life year rate (ASDR) from 1990 to 2021 in 204 countries and territories, stratifying the data by region, country, and sociodemographic index (SDI). These findings provide information on policy implications for policymakers, and how to optimize the allocation of healthcare resources and ultimately alleviate the disease burden.

## 2 METHODS

### 2.1 Data sources

A secondary analysis was performed using data from the 2021 GBD study, which assessed health losses across 371 diseases and injuries and 88 risk factors in 204 countries or territories. The burden of disease refers to the overall socioeconomic and health impacts, resulting in poor health, disability, and premature death. Lung cancer, succinctly defined as tumors within the trachea, bronchus, or lung, is classified in the International Classification of Diseases (ICD) 10 by codes C33 and C34‐C34.92, and in the ICD 9 by codes 162–162.9, 209.21, V10.1‐V10.20, V16.1‐V16.2, and V16.4‐V16.40.^14^ The women of childbearing age group were aged between 15 and 49 years.^15^ Given that no lung cancer cases were observed in individuals under 24 years of age, the population aged 25–49 years with lung cancer was selected for inclusion. To evaluate the burden of lung cancer attributable to household PM2.5, the mortality and DALYs were assessed and calculated. The SDI is an aggregate measure of the social and economic conditions that affect health outcomes in different regions. It is derived from the geometric mean of indices ranging from 0 to 1 for total fertility rate (TFR) for individuals under 25 years (TFU25), the mean education level for those aged 15 years and above (EDU15+), and lag‐distributed income (LDI) per capita. In the GBD 2021 study, after computing the SDI, the values were scaled by 100 to create a scale ranging from 0 to 100.^14^

In this study, the estimates and 95% uncertainty intervals (UIs) for mortality and DALYs were extracted to measure the burden of lung cancer attributable to household PM2.5 among women of childbearing age. Data were extracted from the GBD 2021 study covering the global levels, five development‐level regions, 21 GBD regions, and 204 countries and territories from 1990 to 2021. The cancer data were organized into 5‐year age groups, specifically 25–29, 30–34, 35–39, 40–44, and 45–49 years.

### 2.2 Statistics analysis

Absolute numbers of mortality and DALYs were calculated by summing the corresponding values across all relevant 5‐year age cohorts. The estimates were age‐standardized to lung cancer attributable to household PM2.5 among women of childbearing age using direct age‐standardization. The purpose of age‐standardization is to eliminate the impact of differences in age structure. The ASMR and ASDR were calculated as $\frac{\sum_{i=1}^{A}a_{i}w_{i}}{\sum_{i=1}^{A}w_{i}}$×100,000 population, where $a_{i}$ denotes the age‐specific rate of the indicators for the *i‐th* age group, $w_{i}$ represents the weights for the *i‐th* age group calculated according to the GBD 2021 world standard population, and *A* is the total number of age groups, which is 5.^16^

The Joinpoint regression model was used to analyze the temporal trends of the above indicators for the burden of lung cancer attributable to household PM2.5 among women of childbearing age over the period from 1990 to 2021. The estimated relationships among the ASDR, ASMR, and SDI were assessed using Spearman's correlation analysis. Age‐standardized rate (ASR) values were reported per 100,000 people, with data presented as values with 95% UI. Temporal trends were assessed using Joinpoint software (version 5.0.2) provided by the National Cancer Institute. All statistical analyses and mapping were performed using the R statistical software (version 4.3.3). A two‐sided *p* value <0.05 was considered the significance threshold.

## 3 RESULTS

### 3.1 Global burden trends

**FIGURE 1:**
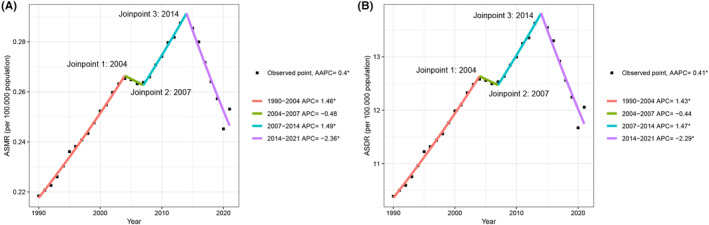
Joinpoint regression analysis for the ASMR (A) and ASDR (B) of global lung cancer attributable to ambient PM2.5 among women of childbearing age from 1990 to 2021. AAPC, average annual percentage change; APC, annual percentage change; ASDR, age‐standardized DALYs rate; SMR, age‐standardized mortality rate; DALYs, disability‐adjusted life‐years.

In 2021, the global number of mortality and DALYs among women of childbearing age due to ambient PM2.5‐related lung cancer were 5205 and 247,211, respectively. These figures increased substantially compared with those in 1990. In addition, the ASMR and ASDR have also increased. The ASMR of women of childbearing age in 2021 (ASMR = 0.25 per 100,000 population, 95% UI: 0.14–0.37) was higher compared to 1990 (ASMR = 0.22 per 100,000 population, 95% UI: 0.13–0.33; AAPC = 0.40, 95% confidence interval (CI): 0.11–0.70, *p* = 0.007). Similarly, the ASDR of women of childbearing age in 2021 (ASDR = 12.06 per 100,000 population, 95% UI: 6.83–17.51) was higher compared to 1990 (ASDR = 10.39 per 100,000 population, 95% UI: 5.96–15.72; AAPC = 0.41, 95% CI: 0.11–0.70, *p* = 0.006). Joinpoint regression analysis revealed significant inflection points in the ASMR in 2004, 2007, and 2014. The ASMR increased between 1990 and 2004 (APC = 1.46, 95% CI: 1.32–1.59, *p* < 0.001), decreased at a relatively slow rate between 2004 and 2007 (APC = −0.48, 95% CI: −3.23 to 2.36, *p* = 0.727), continued to increase between 2007 and 2014 (APC = 1.49, 95% CI: 1.01–1.98, *p* < 0.001), and declined dramatically between 2014 and 2021 (APC = −2.36, 95% CI: −2.73 to −1.99, *p* < 0.001). The ASDR of ambient PM2.5‐related lung cancer in women of childbearing age followed a similar pattern (Figure 1 and Table 1).

**TABLE 1:** Mortality and DALYs of lung cancer attributable to ambient PM2.5 among women of childbearing age in 1990 and 2021, and the AAPC from 1990 to 2021 by SDI quintile and region level.

Location	Mortality					DALYs				
	Mortality cases in 1990 (95% UI)	ASMR in 1990 (95% UI)	Mortality cases in 2021 (95% UI)	ASMR in 2021 (95% UI)	AAPC% (95%CI), 1990–2021	DALYs cases in 1990 (95% UI)	ASDR in 1990 (95% UI)	DALYs cases in 2021 (95% UI)	ASDR in 2021 (95% UI)	AAPC% (95%CI), 1990–2021
Global	2434 (1398–3683)	0.22 (0.13–0.33)	5205 (2947–7562)	0.25 (0.14–0.37)	0.40 (0.11 to 0.70)	116,993 (67,093–177,262)	10.39 (5.96–15.72)	247,211 (140,075–359,108)	12.06 (6.83–17.51)	0.41 (0.11 to 0.70)
25–29 years	89 (50–141)	0.04 (0.02–0.06)	141 (82–208)	0.05 (0.03–0.07)	0.55 (0.13 to 0.97)	5665 (3162–8897)	2.57 (1.43–4.04)	37,462 (21,246–54,185)	3.06 (1.78–4.51)	0.54 (0.12 to 0.96)
30–34 years	182 (103–599)	0.09 (0.05–0.15)	376 (213–560)	0.13 (0.07–0.19)	0.85 (0.52 to 1.18)	10,613 (6032–16,535)	5.58 (3.17–8.69)	66,000 (37491–94,913)	7.30 (4.13–10.85)	0.82 (0.34 to 1.30)
35–39 years	388 (222–599)	0.22 (0.13–0.34)	703 (399–1018)	0.25 (0.14–0.36)	0.31 (0.13 to 0.49)	20,648 (11,825–31,846)	11.90 (6.81–18.36)	112,990 (63,777–164,442)	13.48 (7.64–19.50)	0.31 (0.15 to 0.48)
40–44 years	694 (395–1035.77)	0.49 (0.28–0.74)	1369 (777–1971)	0.55 (0.31–0.79)	0.25 (−0.27 to 0.78)	33,482 (19,058–49,928)	23.87 (13.59–35.60)	8928 (5189–13,125)	26.60 (15.11–38.25)	0.24 (−0.28 to 0.77)
45–49 years	1077 (625–1621)	0.94 (0.55–1.42)	2612 (1474–3802)	1.11 (0.63–1.61)	0.46 (−0.01 to 0.94)	46,584 (27,014–70,054)	40.93 (23.73–61.55)	21,829 (12,370–32,440)	47.95 (27.06–69.78)	0.46 (0 to 0.92)
High SDI	845 (476–1325)	0.36 (0.2–0.56)	473 (288–688)	0.16 (0.1–0.23)	−2.67 (−2.85 to −2.48)	39,841 (22,506–62,326)	16.83 (9.5–26.34)	22,315 (13,554–32,401)	7.47 (4.54–10.84)	−2.63 (−2.79 to −2.47)
High‐middle SDI	781 (427–1249)	0.32 (0.17–0.51)	1830 (1038–2728)	0.47 (0.26–0.69)	1.22 (0.93 to 1.51)	37,725 (20,560–60,437)	15.25 (8.32–24.37)	86,313 (49,011–128,742)	22.23 (12.63–33.15)	1.22 (0.94 to 1.51)
Middle SDI	663 (366–1079)	0.19 (0.11–0.31)	2285 (1242–3373)	0.33 (0.18–0.49)	1.71 (1.55 to 1.88)	32,367 (17,856–52,710)	9.25 (5.1–15.05)	108,690 (59,105–160,365)	15.82 (8.61–23.33)	1.70 (1.53 to 1.87)
Low‐middle SDI	124 (72–191)	0.06 (0.03–0.09)	541 (305–843)	0.12 (0.07–0.18)	2.25 (1.88 to 2.62)	6045 (3510–9294)	2.8 (1.63–4.3)	26,201 (14,803–40,923)	5.62 (3.17–8.77)	2.21 (1.81 to 2.60)
Low SDI	18 (10–29)	0.02 (0.01–0.03)	72 (41–116)	0.03 (0.02–0.06)	1.43 (0.95 to 1.90)	854 (491–1389)	1.02 (0.59–1.66)	3526 (2007.48–5642)	1.67 (0.95–2.66)	1.45 (0.97 to 1.92)
Andean Latin America	23 (9–41)	0.31 (0.13–0.56)	39 (20–66)	0.23 (0.12–0.38)	−0.94 (−1.93 to 0.06)	1118 (454–2012)	15.16 (6.16–27.26)	1897 (957–3193)	11 (5.55–18.51)	−0.96 (−1.95 to 0.04)
Australasia	5 (0–15)	0.09 (0–0.27)	7 (3–12)	0.08 (0.04–0.14)	−0.03 (−0.67 to 0.61)	234 (7–685)	4.32 (0.13–12.61)	338 (159–560)	3.95 (1.85–6.55)	−0.01 (−0.65 to 0.62)
Caribbean	16 (5–33)	0.21 (0.06–0.44)	22 (10–39)	0.18 (0.08–0.31)	−0.50 (−1.16 to 0.16)	747 (226–1567)	9.85 (2.98–20.66)	1056 (469–1817)	8.45 (3.75–14.55)	−0.50 (−1.14 to 0.15)
Central Asia	29 (11–56)	0.23 (0.08–0.45)	42 (24–64)	0.17 (0.1–0.25)	−0.99 (−1.61 to −0.37)	1446 (535–2828)	11.09 (4.1–21.66)	2066 (1191–3113)	8.13 (4.69–12.25)	−0.99 (−1.61 to −0.38)
Central Europe	142 (63–237)	0.44 (0.2–0.73)	100 (61–143)	0.28 (0.17–0.4)	−1.71 (−2.03 to −1.39)	6717 (2980–11,167)	20.73 (9.2–34.47)	4648 (2809–6619)	13.16 (7.96–18.75)	−1.72 (−2.04 to −1.39)
Central Latin America	70 (34–114)	0.23 (0.11–0.37)	71 (41–106)	0.1 (0.06–0.15)	−2.53 (−2.99 to −2.07)	3424 (1657–5596)	10.9 (5.28–17.82)	3389 (1960–5071)	4.83 (2.8–7.23)	−2.54 (−3.00 to −2.08)
Central Sub‐Saharan Africa	2 (1–5)	0.03 (0.01–0.05)	11 (5–21)	0.05 (0.02–0.08)	1.89 (1.71 to 2.07)	110 (51–217)	1.24 (0.57–2.44)	548 (246–991)	2.21 (0.99–4)	1.90 (1.73 to 2.08)
East Asia	776 (344–1507)	0.29 (0.13–0.57)	2926 (1502–4497)	0.68 (0.35–1.04)	2.69 (2.45 to 2.93)	37,932 (16,790–73,622)	14.03 (6.22–27.26)	138,493 (71,120–212,665)	32.45 (16.65–49.8)	2.68 (2.45 to 2.92)
Eastern Europe	175 (81–282)	0.32 (0.15–0.51)	68 (36–111)	0.11 (0.06–0.17)	−3.6 (−4.07 to −3.17)	8406 (3877–13,519)	14.99 (6.92–24.1)	3241 (1685–5297)	5.08 (2.64–8.3)	−3.60 (−4.05 to −3.14)
Eastern Sub‐Saharan Africa	4 (2–6)	0.01 (0.01–0.02)	16 (8–26)	0.02 (0.01–0.03)	1.45 (1.03 to 1.88)	187 (105–306)	0.63 (0.36–1.03)	762 (389–1268)	0.97 (0.5–1.61)	1.47 (1.05 to 1.89)
High‐income Asia Pacific	110 (31–223)	0.22 (0.06–0.44)	69 (37–109)	0.13 (0.07–0.2)	−1.79 (−2.24 to −1.34)	5297 (1528–10,737)	10.65 (3.1–21.56)	3243 (1757–5152)	6.16 (3.32–9.8)	−1.82 (−2.26 to −1.37)
High‐income North America	322 (125–583)	0.43 (0.17–0.77)	52 (22–91)	0.05 (0.02–0.09)	−6.25 (−6.65 to −5.85)	14,989 (5807–27,145)	19.75 (7.65–35.77)	2418 (1018–4218)	2.51 (1.06–4.37)	−6.24 (−6.62 to −5.86)
North Africa and Middle East	108 (61–166)	0.18 (0.1–0.29)	383 (233–546)	0.24 (0.15–0.34)	0.78 (0.49 to 1.06)	5294 (3010–8182)	8.91 (5.07–13.78)	18,573 (11,293–26,479)	11.6 (7.05–16.53)	0.78 (0.50 to 1.06)
Oceania	0 (0–1)	0.03 (0.01–0.1)	2 (0–4)	0.05 (0.02–0.13)	1.35 (0.97 to 1.74)	19 (5–55)	1.64 (0.4–4.75)	77 (23–194)	2.48 (0.76–6.25)	1.36 (0.98 to 1.74)
South Asia	74 (38–127)	0.04 (0.02–0.06)	559 (313–865)	0.12 (0.07–0.19)	3.98 (3.39 to 4.57)	3613 (1840–6208)	1.74 (0.89–3)	26,876 (15,042–41,654)	5.82 (3.26–9.02)	3.96 (3.36 to 4.56)
Southeast Asia	146 (60–281)	0.16 (0.07–0.31)	513 (261–812)	0.26 (0.13–0.41)	1.57 (1.34 to 1.80)	7104 (2898–13,624)	7.65 (3.13–14.68)	24,444 (12,502–38,635)	12.54 (6.42–19.81)	1.58 (1.34 to 1.82)
Southern Latin America	28 (12–52)	0.24 (0.1–0.44)	32 (15–53)	0.17 (0.08–0.28)	−1.21 (−1.54 to −0.88)	1347 (565–2460)	11.45 (4.81–20.91)	1497 (725–2516)	7.9 (3.83–13.28)	−1.15 (−1.49 to −0.81)
Southern Sub‐Saharan Africa	21 (11–32)	0.22 (0.12–0.33)	34 (19–53)	0.17 (0.09–0.26)	−0.95 (−1.43 to −0.47)	1033 (558–1563)	10.47 (5.67–15.82)	1627 (889–2545)	8.02 (4.39–12.51)	−1.05 (−1.55 to −0.54)
Tropical Latin America	44 (14–88)	0.14 (0.04–0.28)	84 (41–134)	0.12 (0.06–0.2)	−0.27 (−0.75 to 0.22)	2107 (686–4279)	6.49 (2.12–13.19)	3981 (1962–6324)	5.91 (2.92–9.39)	−0.29 (−0.78 to 0.20)
Western Europe	334 (148–566)	0.33 (0.14–0.56)	153 (87–230)	0.12 (0.07–0.19)	−3.19 (−3.41 to −2.98)	15,626 (6912–26,447)	15.35 (6.79–25.98)	7087 (4033–10,628)	5.82 (3.31–8.74)	−3.21 (−3.44 to −2.99)
Western Sub‐Saharan Africa	5 (3–8)	0.02 (0.01–0.03)	19 (9–36)	0.02 (0.01–0.04)	0.87 (0.21 to 1.53)	245 (128–405)	0.8 (0.42–1.32)	949 (457–1749)	1.05 (0.51–1.94)	0.89 (0.23 to 1.56)

*Note*: The rates are reported per 100,000 people per year. Data in parentheses are 95% uncertainty intervals for cases and age‐standardized rates of mortality and DALYs, and 95% confidence intervals for AAPCs.

Abbreviations: AAPC, average annual percent change; ASDR, age‐standardized DALYs rate; ASMR, age‐standardized mortality rate; CI, confidence interval; DALYs, disability‐adjusted life‐years; SDI, socio‐demographic index; UI, uncertainty interval.

### 3.2 Global burden trends by age group

Globally, there was a consistent increase in age‐specific DALY and death rates across all age groups from 1990 to 2021; the largest increase in ASDR between 1990 and 2021 was noted in those aged 30–34 years (AAPC = 0.82, 95% CI: 0.34–1.30, *p =* 0.001). The ASDR in those aged 30–34 years increased from 5.58 per 100,000 population in 1990 to 7.30 per 100,000 population in 2021. Similarly, individuals aged 30–34 years experienced the largest increase in ASMR between 1990 and 2021 (AAPC = 0.85, 95% CI: 0.52–1.18, *p* < 0.001). No data were available on the ASMR and ASDR in women of childbearing age aged 15–19 years and 20–24 years. Furthermore, no statistically significant change was observed in ASMR among women of childbearing age aged 40–44 years (AAPC = 0.25, 95% CI: −0.27 to 0.78, *p* = 0.35) and 45–49 years (AAPC = 0.46, 95% CI: −0.01 to 0.94, *p =* 0.055). Similarly, no statistically significant changes were observed in the ASDR among women of childbearing age aged 40–44 years (AAPC = 0.24, 95% CI: −0.28 to 0.77, *p* = 0.36). In 2021, those aged 35–39 years accounted for 112,990 (45.7%) of the 247,211 DALYs cases of ambient PM2.5‐related lung cancer among women of childbearing age. In both 2021 and 1990, the crude DALY and death rates of ambient PM2.5‐related lung cancer among women of childbearing age increased with age and peaked in the 45–49 years age group (Table 1).

### 3.3 Burden trends by region and country

**FIGURE 2:**
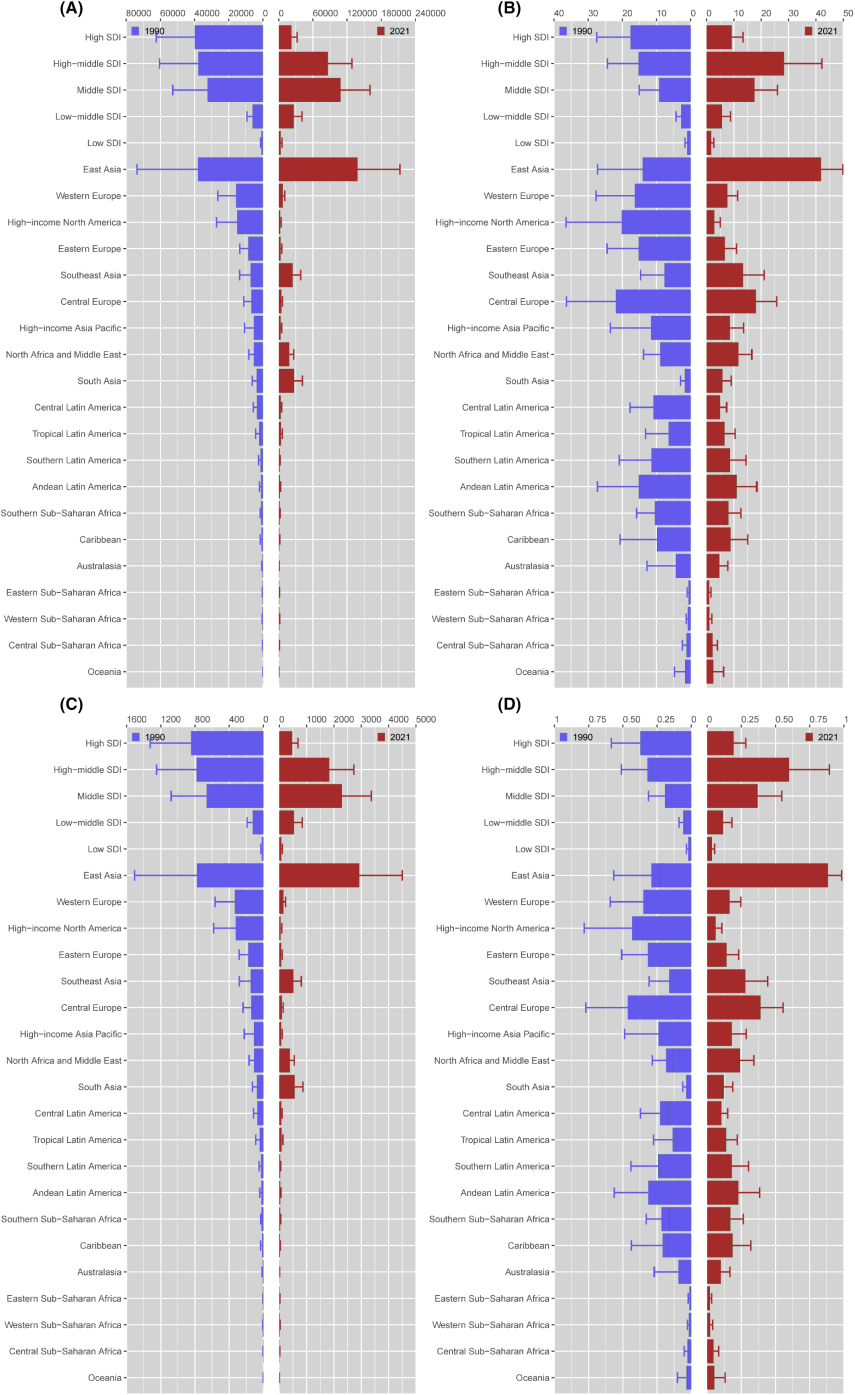
Change in the burden of global lung cancer attributable to ambient PM2.5 among women of childbearing age by region, 1990 versus 2021. Columns and error bars represent the central estimates and 95% uncertainty interval of DALYs (A), ASDR (B), mortality (C), and ASMR (D) among women of childbearing age. ASDR, age‐standardized DALYs rate; ASMR, age‐standardized mortality rate; DALYs, disability‐adjusted life‐years; SDI, sociodemographic index.

At the regional level, East and South Asia exhibited the most significant increases in ASMR and ASDR from 1990 to 2021, with the corresponding AAPCs both appearing to be greater than 2. High‐income North America witnessed the most rapid decline in ASMR and ASDR. The AAPC for ASMR was −6.25 (95% Cl: −6.65 to −5.85, *p* < 0.001), whereas the AAPC for ASDR was −6.24 (95% Cl: −6.62 to −5.86, *p* < 0.001). In 1990, Central Europe and High‐income North America reported the highest ASMR (Central Europe: 0.44 per 100,000 population, 95% UI: 0.20–0.73; High‐income North America: 0.43 per 100,000 population, 95% UI: 0.17–0.77) and ASDR (Central Europe: 20.73 per 100,000 population, 95% UI: 9.20–34.47; High‐income North America: 19.75 per 100,000 population, 95% UI: 7.65–35.77). In 2021, High‐income North America showed a decline in the ASMR and ASDR over time; in contrast, East and Southeast Asia emerged as the regions with the highest ASMR and ASDR. Therefore, East Asia, South Asia, and Southeast Asia, the three leading regions for the ambient PM2.5‐related lung cancer burden among women of childbearing age, accounted for over 76% of the global deaths and DALYs (Figure 2 and Table 1).

**FIGURE 3:**
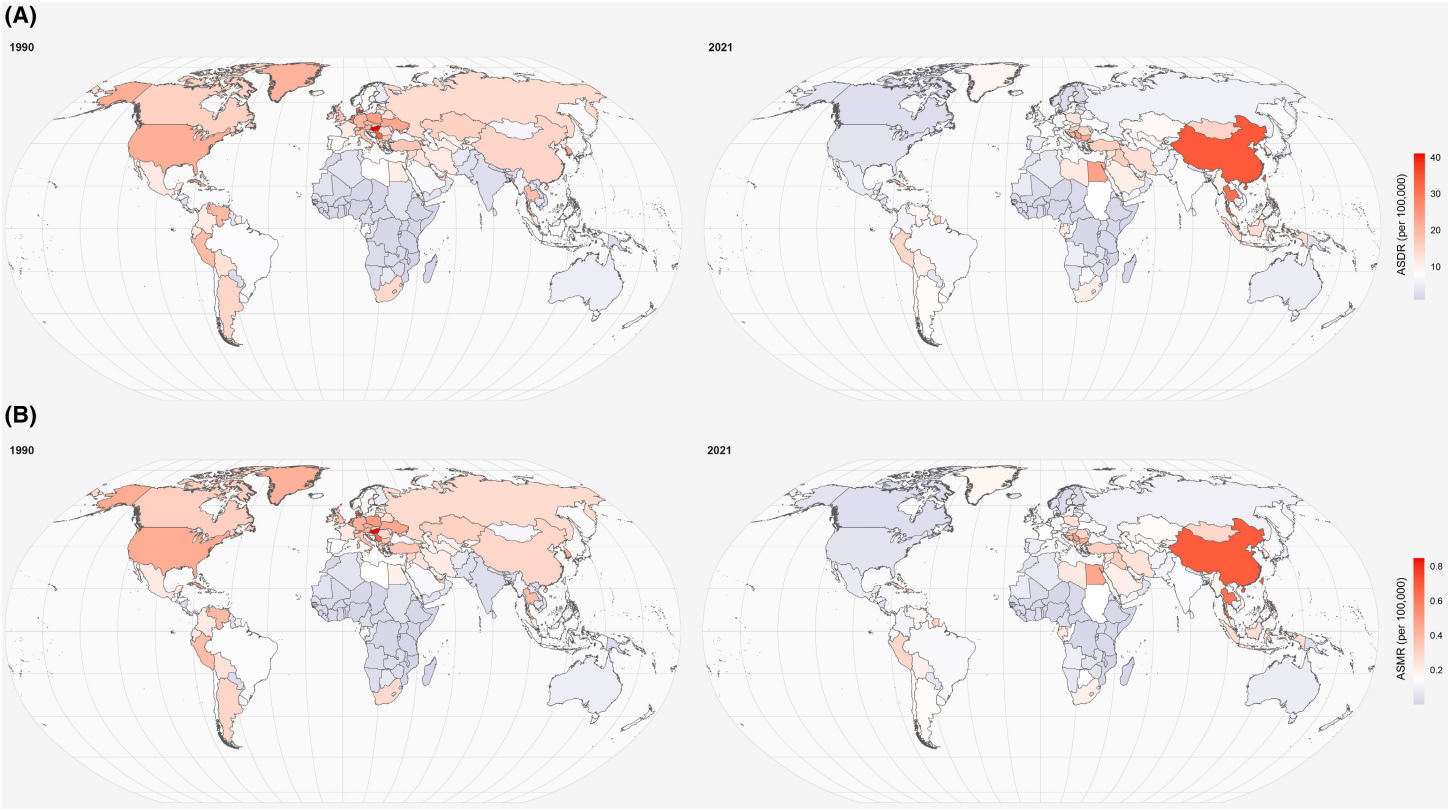
The global distribution of the ASDR (A) and ASMR (B) of lung cancer attributable to ambient PM2.5 among women of childbearing age in 1990 and 2021. ASDR, age‐standardized DALYs rate; ASMR, age‐standardized mortality rate; DALYs, disability‐adjusted life years.

At the national level, the most pronounced increases in the ASMR and ASDR between 1990 and 2021 were observed in Equatorial Guinea, Bhutan, and Vietnam, with the most pronounced decreases observed in Canada, the United States of America, and Estonia. Notably, the top three countries with the highest ASMR and ASDR were China, Thailand, and Serbia in 2021, whereas Hungary, Denmark, and Montenegro had the highest ASMR and ASDR in 1990. Simultaneously, the top three countries with the heaviest burdens in the world shifted from China, the United States of America, and the Russian Federation in 1990 to China, India, and Indonesia in 2021 (Figure 3 and Table S1).

### 3.4 Burden trends associated with the SDI

Among the SDI regions, the only decreases in ASMR and ASDR were noted in the high SDI region, whereas, the low‐middle SDI, middle SDI, low SDI, and high‐middle SDI regions all exhibited increasing trends from 1990 to 2021. Notably, in terms of ambient PM2.5‐related lung cancer among women of childbearing age, the high‐middle SDI region was identified as the leading region for the ASMR and ASDR in 2021, with rates of 0.47 and 22.23 cases per 100,000 population, respectively. Conversely, the high SDI region had the highest ASMR and ASDR in 1990. In contrast, the low SDI region has the lowest ASMR and ASDR in 1990 and 2021. Additionally, the middle SDI region has the highest number of mortality and DALYs in 2021, indicating that it has the highest burden. However, in 1990, the high SDI region had the heaviest burden (Figure 2 and Table 1).

**FIGURE 4:**
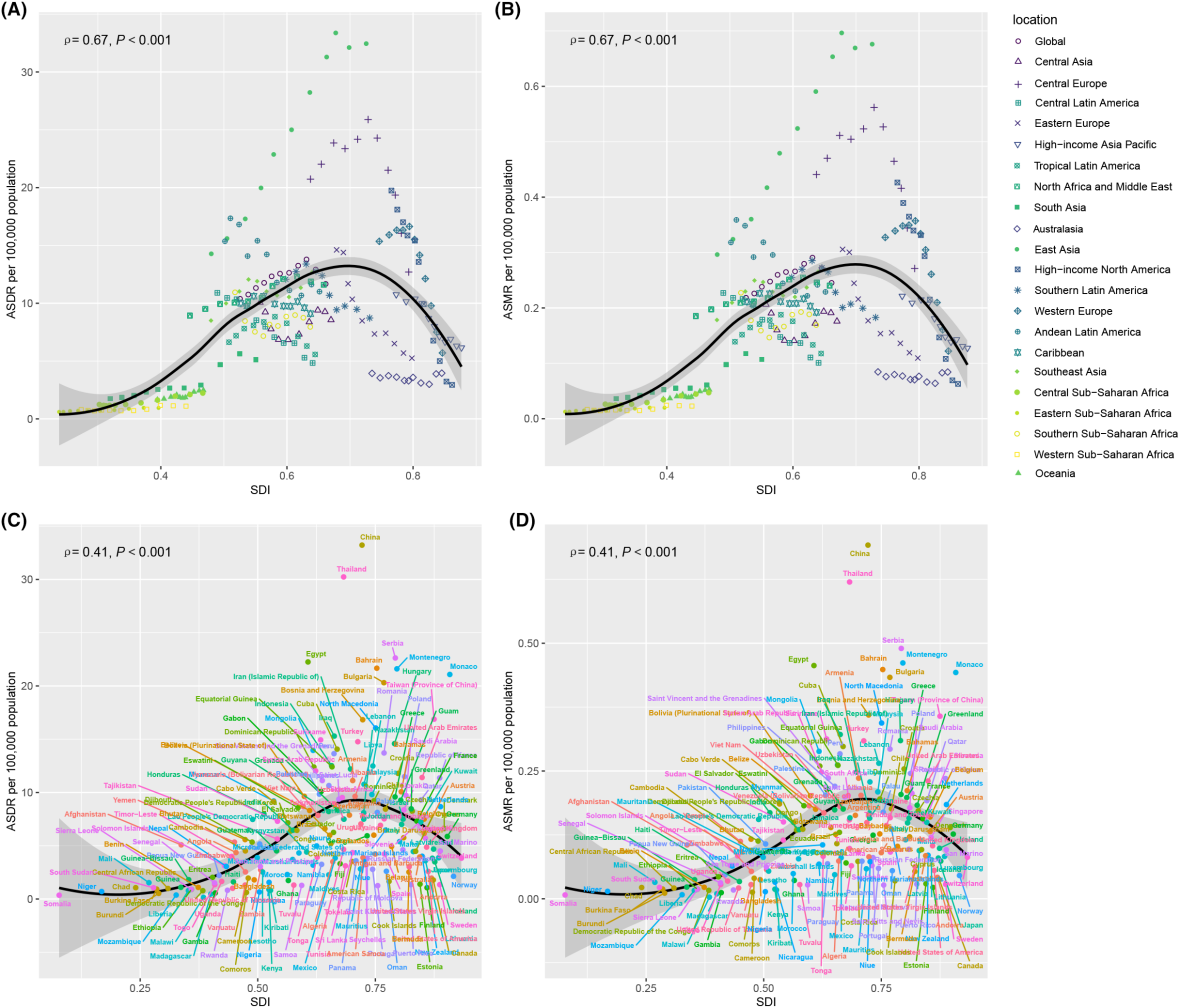
Correlations between the ASDR and ASMR of lung cancer attributable to ambient PM2.5 among women of childbearing age and SDI at the regional and national level. ASDR of lung cancer attributable to ambient PM2.5 among women of childbearing age at the global level and 21 regions by SDI from 1990 to 2021 (A). ASMR of lung cancer attributable to ambient PM2.5 among women of childbearing age at the global level and 21 regions by SDI from 1990 to 2021 (B). ASDR of lung cancer attributable to ambient PM2.5 among women of childbearing age in 204 countries or territories by SDI in 2021 (C). ASMR of lung cancer attributable to ambient PM2.5 among women of childbearing age in 204 countries or territories by SDI in 2021 (D). ASDR, age‐standardized DALYs rate; ASMR, age‐standardized mortality rate; DALYs, disability‐adjusted life years; SDI, sociodemographic index.

**FIGURE 5:**
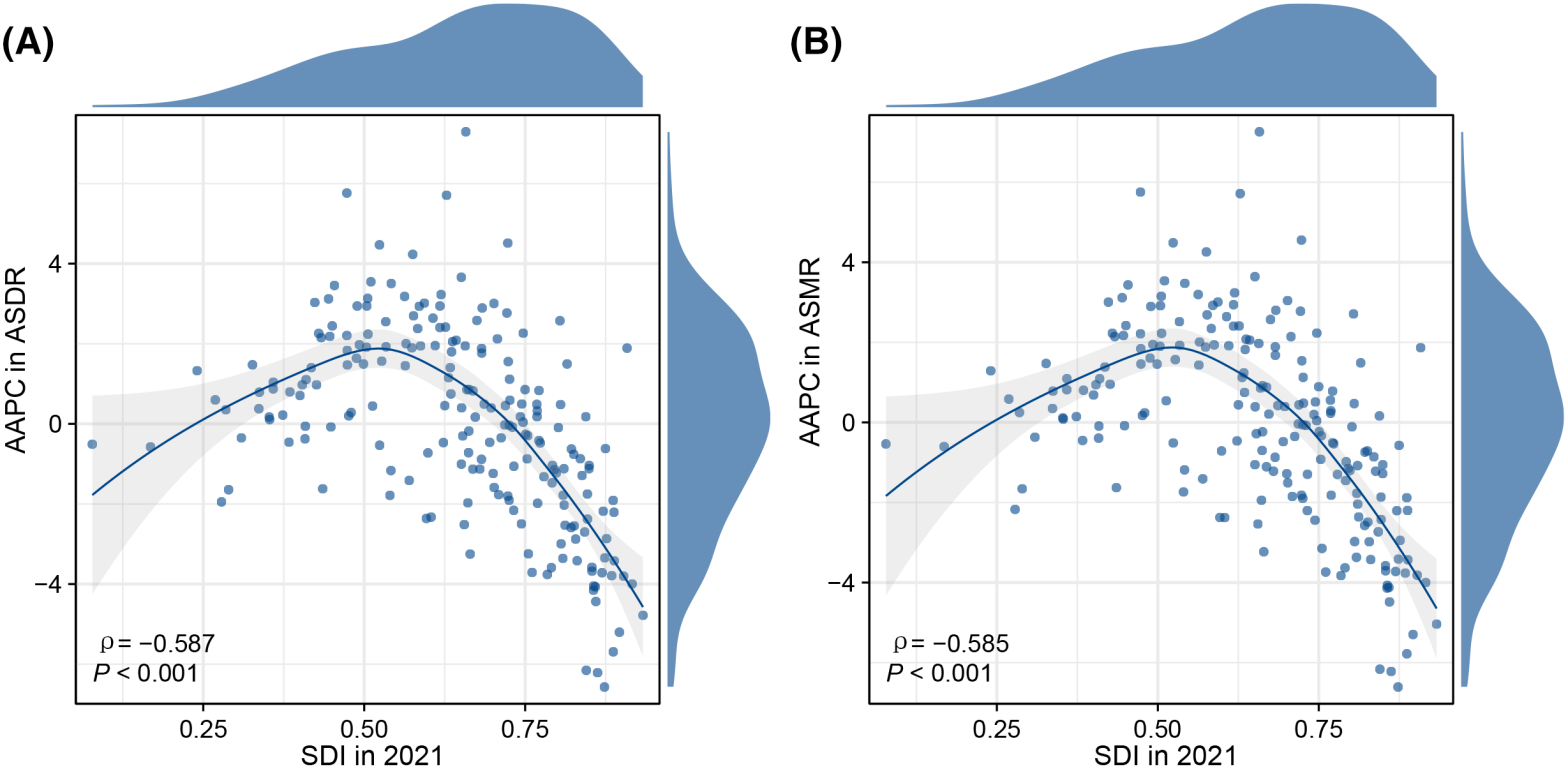
AAPC of the ASDR (A) and ASMR (B) from 1990 to 2021 in 204 countries and territories according to the SDI in 2021. AAPC, average annual percentage change; ASDR, age‐standardized DALYs rate; ASMR, age‐standardized mortality rate; DALYs, disability‐adjusted life years; SDI, sociodemographic index.

Significant positive correlations were found between SDI and both ASMR and ASDR regarding the ambient PM2.5‐related lung cancer burden among women of childbearing age. At the regional and national levels, the ASDR and ASMR increased with SDI, but decreased substantially at higher SDI levels (Figure 4). Similar patterns were observed between the SDI and the AAPC of the ASMR and ASDR (Figure 5). At the regional level, the observed global ASMR and ASDR slightly exceeded the fitted curve. Moreover, East Asia and Central Europe exhibited higher observed values than the fitted curves, and China and Thailand exhibited significantly higher ASMR and ASDR than the fitted curves.

## 4 DISCUSSION

Previous GBD analyses have focused on the lung cancer burden among the whole global population or in specific regions, while analyses on the lung cancer burden due to specific risk factors or affecting specific groups of the population are often neglected. To the best of our knowledge, our study is the first to report on the global lung cancer burden attributable to ambient PM2.5 among women of childbearing age using data from the post‐COVID‐19 pandemic period, including comparisons across regions, countries, and age groups. This study provides the most comprehensive and detailed assessment of the global burden of ambient PM2.5‐related lung cancer in women of childbearing age from 1990 to 2021. An upward trend was observed in the global burden of lung cancer attributable to ambient PM2.5 among women of childbearing age. The lung cancer burden exhibited significant regional heterogeneity, which is associated with socioeconomic factors. The middle SDI region, East Asia, and China exhibited the highest lung cancer burden attributable to ambient PM2.5 exposure. The ASMR and ASDR of lung cancer attributable to ambient PM2.5 exhibited the most rapid decline in the high SDI region, high‐income North America, Canada, and the United States of America. Concurrently, an inverted U‐shaped curvilinear relationship was observed between SDI and lung cancer attributable to ambient PM2.5 among women of childbearing age.

Smoking is widely recognized as the primary risk factor for lung cancer development. Following the implementation of the World Health Organization (WHO) Framework Convention on Tobacco Control, smoking rates, and the smoking‐related lung cancer incidence have generally decreased in most countries and territories in recent years.^2^, ^17^ Compared with lung cancer in smokers, lung cancer in never‐smokers (LCINS) is characterized by distinct clinical and molecular features.^18^ LCINS is often an adenocarcinoma carrying epidermal growth factor receptor (EGFR) gene mutations and is characterized by a higher prevalence in Asians,^19^ females, and is associated with radon exposure, occupational carcinogen exposure, and air pollution.^20^ However, in recent years, an increasing trend in the lung cancer incidence has been observed among nonsmoking women, particularly among younger women.^13^, ^21^ As a result, ambient PM2.5 has emerged as the foremost risk factor for lung cancer mortality and DALYs among women of childbearing age. Environmental particulate matter pollution, specifically ambient fine particulate matter (PM2.5 and PM10), has been identified as a critical driver of the increasing incidence of lung cancer, particularly among nonsmokers.^22^, ^23^

Among women of childbearing age, we observed a global increase in the lung cancer tendency in ASMR and ASDR attributable to ambient PM2.5, from 1990 to 2021, in addition to an increase in the number of mortality and DALYs. As of 2019, 99% of the global population resided in environments that did not comply with the WHO air quality guidelines. Increasing evidence indicates that air pollution exacerbates the risk of lung cancer.^24^, ^25^ Consequently, the WHO updated its global air quality guidelines in 2021, recommending that the annual average concentration of PM2.5 should not exceed 5 μg/m^3^.^26^ The ASDR and ASMR of the lung cancer burden attributable to ambient PM2.5 among women of childbearing age peaked in 2014 and subsequently exhibited a gradual decline. This phenomenon may be related to public health policies. In 2013, the International Agency for Research on Cancer (IARC) classified air pollution as a carcinogenic substance. Within polluted air, nitrogen dioxide, sulfur dioxide, ozone, and particulate matter with diameters <10 μm (PM10) and PM2.5 have been identified as carcinogenic.^27^ An analysis of the data post‐COVID‐19 revealed that during the peak of the COVID‐19 pandemic in 2020, a sharp decline in the global burden was observed, followed by a sharp rise in 2021. Although this fluctuation was not directly associated with COVID‐19, it partly reflected the impact of the pandemic on global lung cancer care systems. The reduction in 2020 was linked to the strain on medical resources, delays in cancer screening, and treatment disruptions at the onset of COVID‐19.^28^, ^29^ On the contrary, it reflected the impact of the pandemic on the global air quality.^30^ Due to the implementation of prevention and control measures during the COVID‐19 pandemic and the emergence of the global economic crisis, there was observed a significant decrease in air pollutant concentrations across various cities.^31^ In 2021, with the rollout of vaccinations, optimization of epidemic control measures, and the resumption of healthcare services, an increase in air pollutant concentrations was observed. This is indicative of the positive impact of public health interventions in restoring routine medical services.^32^

Socioeconomic status has been observed to play a moderating role in the impact of ambient PM2.5 on lung cancer among women of childbearing age. Regions with high SDI have shown considerable improvements in reducing the ASMR and ASDR of lung cancer attributable to ambient PM2.5. However, the global ASMR and ASDR continue to increase because the burden is primarily concentrated within the remaining four SDI regions. In addition, the burden of lung cancer attributable to ambient PM2.5 exhibited an inverted U‐shaped relationship with the SDI.^4^, ^15^ This phenomenon may be explained by the fact that ambient PM2.5 exposure increased in low‐to‐middle SDI regions but decreased in high SDI regions, reflecting an inverted U‐shaped relationship between ambient PM2.5 and socioeconomic development.

At the regional level, regions with a high SDI typically implement stricter environmental regulatory policies and maintain better air quality monitoring systems, along with more resources and advanced technologies to reduce pollutant emissions, such as the use of clean energy and improved industrial filtration technologies, which can effectively control ambient PM2.5. For example, the United States, through the expansion of regulatory measures under the Clean Air Act, has achieved significant reductions in the lung cancer burden attributable to environmental particulate matter pollution.^33^ Our findings demonstrate that high SDI countries or regions, such as high‐income North America, Canada, and the United States, have exhibited the most significant declines. Globalization and rapid economic development have changed many occupational exposures and environmental factors. This shift was more evident in regions with middle‐ and middle‐high SDI. Regions with middle and middle‐high SDI may be undergoing rapid industrialization and urbanization, which often leads to high pollutant emissions.^34^, ^35^ Due to limitations in resources and technology, ambient PM2.5 concentrations in these regions tend to be higher. Furthermore, these regions may lack stringent environmental regulatory policies or face challenges in policy enforcement, making it difficult to effectively control ambient PM2.5. Our study indicates that East Asia and Southeast Asia have emerged as the regions with the highest ASMR and ASDR. The 2022 World Air Quality Report released by the World Meteorological Organization (WMO) indicated that most of the 40 most‐polluted cities globally are situated in South Asia. On the one hand, countries in South Asia have long been affected by global warming and extreme natural disasters, which exacerbate ambient PM2.5.^36^ On the other hand, due to limited economic conditions, controlling ambient PM2.5 is not likely to be a high priority. Unchecked industrial emissions, agricultural waste burning, vehicle exhaust emissions, and cremation in some areas all contribute to the worsening of ambient PM2.5.^37^, ^38^, ^39^ Additionally, genetic mutations are considered to play an important role. It has been established that PM2.5 exposure levels are associated with *EGFR*‐driven lung cancer. Inhaled PM2.5 induces local responses in the lungs, which are mediated by macrophages and lung epithelial cells. Macrophages release the inflammatory factor IL‐1β, a key molecule in tumor formation, which promotes PM2.5‐mediated *EGFR*‐driven lung adenocarcinoma (LUAD).^8^, ^20^ Consequently, ambient PM2.5 has been identified as a risk factor for LUAD in individuals with *EGFR* mutations.^8^ Genetic mutations are found in 90% of nonsmoking female lung adenocarcinoma patients, with *EGFR* mutations being the most common. In addition, *EGFR* mutations are especially prevalent among Asian women.^40^ Consequently, East Asia and South Asia have emerged as regions with the heaviest global burden.

At the national level, China has identified as bearing the heaviest burden. China is widely regarded as a middle‐high SDI country with a large population. Economic globalization and rapid urbanization have resulted in significant industrial emissions and traffic pollution in China. Additionally, the widespread use of computed tomography (CT) in China has resulted in an increased detection rate of ambient PM2.5‐related lung cancer among women of childbearing age.^41^ Particularly since the onset of the COVID‐19 pandemic, such cases have often been discovered during medical consultations prompted by typical respiratory symptoms, including coughing, pneumonia, and shortness of breath.^12^ While there is no direct relationship between ambient PM2.5 and COVID‐19, high concentrations of PM2.5 can cause respiratory inflammation and impair the immune system, thereby potentially increasing the risk of COVID‐19 infection.^42^, ^43^ Although China previously reported a severe burden of lung cancer attributable to environmental particulate matter pollution,^44^ PM2.5 pollution has been effectively mitigated and public health has improved through measures such as waste gas emission control programs, reduction of coal use, and industrial structure adjustment.^45^, ^46^

The strengths of this secondary analysis include its systematic estimation of the global burden of lung cancer attributable to household PM2.5 among women of childbearing age from 1990 to 2021. However, it is also imperative to recognize the limitations of this study. First, while data from the GBD database are considered high quality, the sources of GBD data are limited, especially in the regions most affected by the COVID‐19 pandemic and in many low‐ and middle‐income countries (LMICs). Moreover, GBD statistical methods rely heavily on modeled data, introducing additional uncertainties in population estimates. Second, the GBD 2021 database does not distinguish between bronchial and lung cancer, and relevant clinical information is absent. Third, this study utilized manual calculations of ASRs for lung cancer among women of childbearing age, which, although eliminating the influence of age structure differences, precluded the derivation of uncertainty intervals. This approach prevents the determination of uncertainty intervals, impeding the full integration of the quality, availability, and statistical uncertainty of the source data into the estimation outcomes. Finally, while this study provides a global perspective on the SDI associated with the lung cancer burden attributable to household PM2.5 among women of childbearing age, it may be disproportionately influenced by data from populous countries. This could result in conclusions that do not directly apply to or reflect the unique circumstances of specific regions.

## 5 CONCLUSIONS

In summary, this study revealed that the global burden of lung cancer due to household PM2.5 among women of childbearing age increased from 1990 to 2021. The global burden changed with socioeconomic development and exhibited an inverted U‐shaped relationship. The middle SDI region, East Asia, and China bear the greatest burdens. Air quality management has a significant environmental impact. Our study aims to provide evidence to support local governments in implementing regulatory changes to improve air quality, ultimately aiding in the control and prevention of lung cancer among women of childbearing age.

## AUTHOR CONTRIBUTIONS

**Ying‐da Song:** Conceptualization (equal); data curation (equal); formal analysis (equal); investigation (equal); methodology (lead); software (equal); validation (equal); visualization (equal); writing – original draft (lead); writing – review and editing (equal). **Ruizhe Wang:** Data curation (equal); formal analysis (equal); investigation (equal); software (equal); validation (equal). **Jia‐xuan Wang:** Formal analysis (equal); investigation (equal); software (equal); validation (equal); visualization (equal); writing – review and editing (equal). **Xun‐wu Tan:** Data curation (equal); formal analysis (equal); investigation (equal); validation (equal). **Jun Ma:** Conceptualization (equal); funding acquisition (lead); project administration (lead); resources (lead); supervision (lead); writing – review and editing (equal).

## FUNDING INFORMATION

This work was supported by the Shanxi Province Natural Science Foundation (202103021224383).

## CONFLICT OF INTEREST STATEMENT

The authors declare that the research was conducted in the absence of commercial or financial relationships that could be construed as a potential conflict of interest.

## ETHICS STATEMENT

The GBD follows the Guidelines for Accurate and Transparent Health Estimates Reporting (GATHER). Since GBD information is entirely anonymized and does not include personal data, this analysis did not require approval from a research ethics committee.

## Supporting information


Table S1.

## Data Availability

The data used in this study came from a public database that everyone can access through the link provided in this article (https://vizhub.healthdata.org/gbd‐results/).

## References

[1] Bray F , Laversanne M , Sung H , et al. Global cancer statistics 2022: GLOBOCAN estimates of incidence and mortality worldwide for 36 cancers in 185 countries. CA Cancer J Clin. 2024;74(3):229‐263. 38572751 10.3322/caac.21834

[2] Ebrahimi H , Aryan Z , Moghaddam SS , et al. Global, regional, and national burden of respiratory tract cancers and associated risk factors from 1990 to 2019: a systematic analysis for the global burden of disease study 2019. Lancet Respir Med. 2021;9(9):1030‐1049. 34411511 10.1016/S2213-2600(21)00164-8 PMC8410610

[3] Chen J , Cui Y , Deng Y , et al. Global, regional, and national burden of cancers attributable to particulate matter pollution from 1990 to 2019 and projection to 2050: worsening or improving? J Hazard Mater. 2024;477:135319. 39059291 10.1016/j.jhazmat.2024.135319

[4] Sang S , Chu C , Zhang T , Chen H , Yang X . The global burden of disease attributable to ambient fine particulate matter in 204 countries and territories, 1990‐2019: a systematic analysis of the global burden of disease study 2019. Ecotoxicol Environ Saf. 2022;238:113588. 35525115 10.1016/j.ecoenv.2022.113588

[5] GBD 2021 Risk Factors Collaborators . Global burden and strength of evidence for 88 risk factors in 204 countries and 811 subnational locations, 1990–2021: a systematic analysis for the Global Burden of Disease Study 2021. Lancet (London, England). 2024;403(10440):2162‐2203. 38762324 10.1016/S0140-6736(24)00933-4 PMC11120204

[6] Newby DE , Mannucci PM , Tell GS , et al. Expert position paper on air pollution and cardiovascular disease. Eur Heart J. 2015;36(2):83‐93b. 25492627 10.1093/eurheartj/ehu458 PMC6279152

[7] GBD 2017 Risk Factor Collaborators . Global, regional, and national comparative risk assessment of 84 behavioural, environmental and occupational, and metabolic risks or clusters of risks for 195 countries and territories, 1990–2017: A systematic analysis for the global burden of disease study 2017. Lancet (London, England). 2018;392(10159):1923‐1994. 30496105 10.1016/S0140-6736(18)32225-6 PMC6227755

[8] Hill W , Lim EL , Weeden CE , et al. Lung adenocarcinoma promotion by air pollutants. Nature. 2023;616(7955):159‐167. 37020004 10.1038/s41586-023-05874-3 PMC7614604

[9] Lo WC , Ho CC , Tseng E , Hwang JS , Chan CC , Lin HH . Long‐term exposure to ambient fine particulate matter (PM2.5) and associations with cardiopulmonary diseases and lung cancer in Taiwan: a nationwide longitudinal cohort study. Int J Epidemiol. 2022;51(4):1230‐1242. 35472171 10.1093/ije/dyac082

[10] Huang Y , Zhu M , Ji M , et al. Air pollution, genetic factors, and the risk of Lung cancer: a prospective study in the UK biobank. Am J Respir Crit Care Med. 2021;204(7):817‐825. 34252012 10.1164/rccm.202011-4063OC

[11] Pallis AG , Syrigos KN . Lung cancer in never smokers: disease characteristics and risk factors. Crit Rev Oncol Hematol. 2013;88(3):494‐503. 23921082 10.1016/j.critrevonc.2013.06.011

[12] Zhang Y , Jheon S , Li H , et al. Results of low‐dose computed tomography as a regular health examination among Chinese hospital employees. J Thorac Cardiovasc Surg. 2020;160(3):824‐831.e824. 31987625 10.1016/j.jtcvs.2019.10.145

[13] Jemal A , Miller KD , Ma J , et al. Higher Lung cancer incidence in young women than young men in the United States. N Engl J Med. 2018;378(21):1999‐2009. 29791813 10.1056/NEJMoa1715907 PMC7717174

[14] GBD 2021 Diseases and Injuries Collaborators . Global incidence, prevalence, years lived with disability (YLDs), disability‐adjusted life‐years (DALYs), and healthy life expectancy (HALE) for 371 diseases and injuries in 204 countries and territories and 811 subnational locations, 1990–2021: A systematic analysis for the Global Burden of Disease Study 2021. Lancet (London, England). 2024;403(10440):2133‐2161. 38642570 10.1016/S0140-6736(24)00757-8 PMC11122111

[15] Sun P , Yu C , Yin L , et al. Global, regional, and national burden of female cancers in women of child‐bearing age, 1990‐2021: analysis of data from the global burden of disease study 2021. EClinicalMedicine. 2024;74:102713. 39050105 10.1016/j.eclinm.2024.102713 PMC11268131

[16] Wen YF , Chen MX , Yin G , et al. The global, regional, and national burden of cancer among adolescents and young adults in 204 countries and territories, 1990‐2019: a population‐based study. J Hematol Oncol. 2021;14(1):89. 34108026 10.1186/s13045-021-01093-3 PMC8191013

[17] Bertollini R , Ribeiro S , Mauer‐Stender K , Galea G . Tobacco control in Europe: a policy review. Eur Respir Rev. 2016;25(140):151‐157. 27246592 10.1183/16000617.0021-2016 PMC9487237

[18] Sun S , Schiller JH , Gazdar AF . Lung cancer in never smokers—a different disease. Nat Rev Cancer. 2007;7(10):778‐790. 17882278 10.1038/nrc2190

[19] Midha A , Dearden S , McCormack R . EGFR mutation incidence in non‐small‐cell lung cancer of adenocarcinoma histology: a systematic review and global map by ethnicity (mutMapII). Am J Cancer Res. 2015;5(9):2892‐2911. 26609494 PMC4633915

[20] Myers R , Brauer M , Dummer T , et al. High‐ambient air pollution exposure among never smokers versus ever smokers with Lung cancer. J Thorac Oncol. 2021;16(11):1850‐1858. 34256112 10.1016/j.jtho.2021.06.015

[21] Ma J , Song YD , Bai XM . Global, regional, and national burden and trends of early‐onset tracheal, bronchus, and lung cancer from 1990 to 2019. Thorac Cancer. 2024;15(8):601‐613. 38303633 10.1111/1759-7714.15227 PMC10928250

[22] Turner MC , Krewski D , Pope CA 3rd , Chen Y , Gapstur SM , Thun MJ . Long‐term ambient fine particulate matter air pollution and lung cancer in a large cohort of never‐smokers. Am J Respir Crit Care Med. 2011;184(12):1374‐1381. 21980033 10.1164/rccm.201106-1011OC

[23] Raaschou‐Nielsen O , Andersen ZJ , Beelen R , et al. Air pollution and lung cancer incidence in 17 European cohorts: prospective analyses from the European study of cohorts for air pollution effects (ESCAPE). Lancet Oncol. 2013;14(9):813‐822. 23849838 10.1016/S1470-2045(13)70279-1

[24] Gharibvand L , Lawrence Beeson W , Shavlik D , et al. The association between ambient fine particulate matter and incident adenocarcinoma subtype of lung cancer. Environ Health. 2017;16(1):71. 28646928 10.1186/s12940-017-0268-7 PMC5483320

[25] IARC Working Group on the Evaluation of Carcinogenic Risks to Humans . Outdoor air pollution. IARC Monogr Eval Carcinog Risks Hum. 2016;109:9‐444. 29905447 PMC7682275

[26] Pérez Velasco R , Jarosińska D . Update of the WHO global air quality guidelines: systematic reviews ‐ an introduction. Environ Int. 2022;170:107556. 36395555 10.1016/j.envint.2022.107556 PMC9720155

[27] Loomis D , Grosse Y , Lauby‐Secretan B , et al. The carcinogenicity of outdoor air pollution. Lancet Oncol. 2013;14(13):1262‐1263. 25035875 10.1016/s1470-2045(13)70487-x

[28] Dinmohamed AG , Visser O , Verhoeven RHA , et al. Fewer cancer diagnoses during the COVID‐19 epidemic in The Netherlands. Lancet Oncol. 2020;21(6):750‐751. 32359403 10.1016/S1470-2045(20)30265-5 PMC7252180

[29] Kutikov A , Weinberg DS , Edelman MJ , Horwitz EM , Uzzo RG , Fisher RI . A war on two fronts: cancer care in the time of COVID‐19. Ann Intern Med. 2020;172(11):756‐758. 32219410 10.7326/M20-1133 PMC7133056

[30] Patel VK , Kuttippurath J , Kashyap R . Increased global cropland greening as a response to the unusual reduction in atmospheric PM₂.₅ concentrations during the COVID‐19 lockdown period. Chemosphere. 2024;358:142147. 38677610 10.1016/j.chemosphere.2024.142147

[31] Latif MT , Purhanudin N , Afandi NZM , et al. In‐depth analysis of ambient air pollution changes due to the COVID‐19 pandemic in the Asian monsoon region. Sci Total Environ. 2024;941:173145. 38768732 10.1016/j.scitotenv.2024.173145

[32] Sharpless NE . COVID‐19 and cancer. Science (New York, NY). 2020;368(6497):1290. 10.1126/science.abd3377 32554570

[33] Nunez Y , Benavides J , Shearston JA , et al. An environmental justice analysis of air pollution emissions in the United States from 1970 to 2010. Nat Commun. 2024;15(1):268. 38233427 10.1038/s41467-023-43492-9 PMC10794183

[34] Adesina JA , Piketh SJ , Qhekwana M , Burger R , Language B , Mkhatshwa G . Contrasting indoor and ambient particulate matter concentrations and thermal comfort in coal and non‐coal burning households at South Africa highveld. Sci Total Environ. 2020;699:134403. 31678873 10.1016/j.scitotenv.2019.134403

[35] Wu D , Zheng H , Li Q , et al. Achieving health‐oriented air pollution control requires integrating unequal toxicities of industrial particles. Nat Commun. 2023;14(1):6491. 37838777 10.1038/s41467-023-42089-6 PMC10576764

[36] De Guzman RB , Malik M , Singh N , Ho‐Fung Loong H , Mohan A . Lung cancer in Asia: the impact of climate change. EClinicalMedicine. 2024;74:102680. 10.1016/j.eclinm.2024.102680 PMC11701854 39764182

[37] Bhadauria V , Parmar D , Ganguly R , Rathi AK , Kumar P . Exposure assessment of PM(2.5) in temple premises and crematoriums in Kanpur, India. Environ Sci Pollut Res Int. 2022;29(25):38374‐38384. 35075564 10.1007/s11356-022-18739-5

[38] Rahman MM , Begum BA , Hopke PK , Nahar K , Thurston GD . Assessing the PM(2.5) impact of biomass combustion in megacity Dhaka, Bangladesh. Environ Pollut. 2020;264:114798. 32559884 10.1016/j.envpol.2020.114798 PMC9581344

[39] Acharya P , Sreekesh S , Kulshrestha U , Gupta G . Characterisation of emission from open‐field burning of crop residue during harvesting period in north‐west India. Environ Monit Assess. 2018;190(11):663. 30345463 10.1007/s10661-018-6999-2

[40] Chen YJ , Roumeliotis TI , Chang YH , et al. Proteogenomics of non‐smoking lung cancer in East Asia delineates molecular signatures of pathogenesis and progression. Cell. 2020;182(1):226‐244.e217. 32649875 10.1016/j.cell.2020.06.012

[41] Wu H , Zhang Y , Hu H , et al. Ground glass opacity featured lung adenocarcinoma in teenagers. J Cancer Res Clin Oncol. 2021;147(12):3719‐3724. 33829316 10.1007/s00432-021-03611-9 PMC8026089

[42] Jiang S , Tang L , Lou Z , et al. The changing health effects of air pollution exposure for respiratory diseases: a multicity study during 2017‐2022. Environ Health. 2024;23(1):36. 38609898 10.1186/s12940-024-01083-1 PMC11015632

[43] Zorn J , Simões M , Velders GJM , et al. Effects of long‐term exposure to outdoor air pollution on COVID‐19 incidence: a population‐based cohort study accounting for SARS‐CoV‐2 exposure levels in The Netherlands. Environ Res. 2024;252(Pt 1):118812. 38561121 10.1016/j.envres.2024.118812

[44] Shaddick G , Thomas ML , Amini H , et al. Data integration for the assessment of population exposure to ambient air pollution for global burden of disease assessment. Environ Sci Technol. 2018;52(16):9069‐9078. 29957991 10.1021/acs.est.8b02864

[45] Du P , Du H , Zhang W , et al. Unequal health risks and attributable mortality burden of source‐specific PM(2.5) in China. Environ Sci Technol. 2024;58(25):10897‐10909. 38843119 10.1021/acs.est.3c08789

[46] Ji D , Li J , Shen G , et al. Environmental effects of China's coal ban policy: results from in situ observations and model analysis in a typical rural area of the Beijing‐Tianjin‐Hebei region, China. Atmos Res. 2022;268:106015.

