# Supplementary material for: Global burden of lung cancer in women of childbearing age attributable to ambient particulate matter pollution: 1990–2021
Source: Cancer Med. 2024 Sep 24;13(18):e70241. doi: 10.1002/cam4.70241 (PMC11420659; doi:10.1002/cam4.70241)
Supplement: Supplementary file 1 — Table S1. [file CAM4-13-e70241-s001.docx]

**Supplemental Table 1. Mortality and DALYs of lung cancer attributable to ambient PM2.5 among women of childbearing age in 1990 and 2021 and AAPC from 1990 to 2021 by country．**

| **Location** | **Mortality** | | | | | **DALYs** | | | | |
| --- | --- | --- | --- | --- | --- | --- | --- | --- | --- | --- |
|  | **Mortality cases in 1990 (95% UI)** | **ASMR in 1990 (95% UI)** | **Mortality cases in 2021 (95% UI)** | **ASMR in 2021 (95% UI)** | **AAPC% (95%CI),**  **1990–2021** | **DALYs cases in 1990 (95% UI)** | **ASDR in 1990 (95% UI)** | **DALYs cases in 2021 (95% UI)** | **ASDR in 2021 (95% UI)** | **AAPC% (95%CI),**  **1990–2021** |
| **Afghanistan** | 1(0 to 4) | 0.08(0.02 to 0.21) | 6(2 to 12) | 0.11(0.04 to 0.22) | 0.78(-0.05,1.62) | 68(19 to 186) | 3.70(1.00 to 10.09) | 281(101 to 584) | 5.08(1.82 to 10.53) | 0.79(-0.05,1.64) |
| **Albania** | 1(0 to 1) | 0.10(0.04 to 0.22) | 1(1 to 2) | 0.20(0.09 to 0.35) | 2.14(1.76,2.53) | 31(12 to 68) | 4.95(1.83 to 10.72) | 66(29 to 113) | 9.63(4.25 to 16.56) | 2.12(1.74,2.50) |
| **Algeria** | 2(1 to 4) | 0.06(0.03 to 0.10) | 9(4 to 16) | 0.07(0.04 to 0.13) | 0.86(0.51,1.20) | 106(50 to 188) | 2.70(1.29 to 4.81) | 420(202 to 742) | 3.50(1.68 to 6.18) | 0.86(0.51,1.21) |
| **American Samoa** | 0(0 to 0) | 0.05(0.00 to 0.19) | 0(0 to 0) | 0.08(0.01 to 0.19) | 1.54(1.16,1.93) | 0(0 to 1) | 2.51(0.00 to 9.02) | 0(0 to 1) | 3.87(0.54 to 8.78) | 1.55(1.17,1.94) |
| **Andorra** | 0(0 to 0) | 0.14(0.04 to 0.27) | 0(0 to 0) | 0.04(0.02 to 0.08) | -3.72(-3.96,-3.49) | 1(0 to 2) | 6.38(2.04 to 12.73) | 1(0 to 1) | 1.96(0.72 to 3.89) | -3.72(-3.95,-3.48) |
| **Angola** | 1(0 to 1) | 0.03(0.01 to 0.07) | 5(2 to 11) | 0.09(0.03 to 0.19) | 3.44(3.06,3.81) | 26(9 to 58) | 1.55(0.57 to 3.44) | 257(86 to 539) | 4.36(1.46 to 9.12) | 3.46(3.07,3.84) |
| **Antigua and Barbuda** | 0(0 to 0) | 0.09(0.02 to 0.21) | 0(0 to 0) | 0.09(0.03 to 0.17) | 0.89(0.36,1.43) | 1(0 to 1) | 4.24(0.85 to 9.76) | 1(0 to 2) | 4.22(1.62 to 7.85) | 0.86(0.33,1.38) |
| **Argentina** | 23(9 to 42) | 0.29(0.12 to 0.53) | 21(9 to 36) | 0.16(0.07 to 0.28) | -1.82(-2.35,-1.28) | 1098(449 to 1999) | 13.94(5.70 to 25.38) | 988(441 to 1732) | 7.74(3.45 to 13.58) | -1.80(-2.35,-1.25) |
| **Armenia** | 3(1 to 5) | 0.36(0.11 to 0.69) | 2(1 to 3) | 0.24(0.13 to 0.34) | -1.51(-2.77,-0.23) | 126(40 to 244) | 17.25(5.42 to 33.48) | 95(54 to 138) | 11.12(6.36 to 16.16) | -1.59(-2.85,-0.32) |
| **Australia** | 4(0 to 12) | 0.09(0.00 to 0.26) | 6(3 to 10) | 0.08(0.04 to 0.14) | 0.16(-0.45,0.78) | 187(6 to 547) | 4.11(0.13 to 12.02) | 284(138 to 466) | 3.95(1.91 to 6.47) | 0.18(-0.43,0.79) |
| **Austria** | 9(4 to 14) | 0.40(0.19 to 0.67) | 3(2 to 5) | 0.13(0.07 to 0.20) | -3.71(-4.27,-3.14) | 404(188 to 672) | 18.86(8.76 to 31.40) | 157(87 to 243) | 6.16(3.42 to 9.54) | -3.68(-4.24,-3.12) |
| **Azerbaijan** | 3(1 to 5) | 0.19(0.06 to 0.40) | 5(2 to 9) | 0.16(0.07 to 0.29) | -0.48(-1.46,0.51) | 129(39 to 274) | 9.05(2.73 to 19.30) | 234(97 to 421) | 7.88(3.26 to 14.14) | -0.46(-1.45,0.54) |
| **Bahamas** | 0(0 to 0) | 0.19(0.03 to 0.43) | 0(0 to 1) | 0.21(0.07 to 0.45) | 0.47(-0.18,1.13) | 5(1 to 12) | 8.85(1.52 to 20.32) | 12(4 to 26) | 10.07(3.29 to 21.59) | 0.48(-0.14,1.11) |
| **Bahrain** | 0(0 to 1) | 0.59(0.33 to 0.95) | 2(1 to 3) | 0.45(0.23 to 0.75) | -0.92(-1.72,-0.11) | 23(13 to 37) | 28.24(15.80 to 45.46) | 75(39 to 124) | 21.67(11.28 to 36.04) | -0.87(-1.66,-0.09) |
| **Bangladesh** | 3(1 to 7) | 0.02(0.01 to 0.04) | 16(7 to 33) | 0.04(0.02 to 0.08) | 1.95(1.10,2.81) | 160(60 to 343) | 0.97(0.36 to 2.06) | 782(322 to 1592) | 1.80(0.74 to 3.65) | 1.97(1.12,2.83) |
| **Barbados** | 0(0 to 0) | 0.12(0.03 to 0.26) | 0(0 to 0) | 0.13(0.05 to 0.23) | 0.05(-0.45,0.54) | 3(1 to 7) | 5.82(1.42 to 12.27) | 5(2 to 10) | 6.00(2.33 to 10.83) | 0.04(-0.44,0.53) |
| **Belarus** | 7(3 to 11) | 0.27(0.13 to 0.44) | 3(1 to 5) | 0.10(0.05 to 0.16) | -3.83(-4.10,-3.56) | 310(147 to 507) | 12.56(5.95 to 20.53) | 138(70 to 218) | 4.93(2.52 to 7.84) | -3.76(-4.03,-3.49) |
| **Belgium** | 12(5 to 20) | 0.47(0.21 to 0.80) | 5(3 to 8) | 0.16(0.09 to 0.25) | -3.58(-4.28,-2.87) | 566(252 to 954) | 22.17(9.91 to 37.41) | 231(127 to 358) | 7.50(4.13 to 11.65) | -3.58(-4.29,-2.87) |
| **Belize** | 0(0 to 0) | 0.09(0.02 to 0.22) | 0(0 to 0) | 0.18(0.06 to 0.34) | 1.92(1.11,2.73) | 1(0 to 3) | 4.38(1.02 to 10.20) | 9(3 to 17) | 8.30(2.85 to 16.09) | 1.95(1.13,2.78) |
| **Benin** | 0(0 to 0) | 0.02(0.01 to 0.04) | 1(0 to 1) | 0.02(0.01 to 0.05) | 0.15(-1.04,1.34) | 8(3 to 16) | 1.05(0.44 to 2.08) | 27(10 to 58) | 1.09(0.43 to 2.34) | 0.22(-0.96,1.42) |
| **Bermuda** | 0(0 to 0) | 0.12(0.00 to 0.32) | 0(0 to 0) | 0.05(0.01 to 0.11) | -2.57(-3.26,-1.88) | 1(0 to 3) | 5.64(0.00 to 15.32) | 0(0 to 1) | 2.53(0.50 to 5.21) | -2.59(-3.27,-1.91) |
| **Bhutan** | 0(0 to 0) | 0.02(0.01 to 0.05) | 0(0 to 0) | 0.12(0.04 to 0.24) | 5.75(5.01,6.50) | 1(0 to 2) | 0.93(0.27 to 2.30) | 11(4 to 22) | 5.58(1.96 to 11.37) | 5.76(5.02,6.51) |
| **Bolivia (Plurinational State of)** | 3(1 to 6) | 0.25(0.09 to 0.52) | 6(2 to 12) | 0.20(0.07 to 0.41) | -0.71(-1.06,-0.36) | 145(51 to 309) | 11.91(4.20 to 25.30) | 281(102 to 571) | 9.64(3.51 to 19.56) | -0.73(-1.07,-0.38) |
| **Bosnia and Herzegovina** | 1(0 to 2) | 0.09(0.04 to 0.17) | 4(1 to 6) | 0.36(0.15 to 0.58) | 4.56(3.83,5.29) | 46(22 to 89) | 4.19(1.98 to 8.13) | 162(68 to 261) | 16.83(7.09 to 27.13) | 4.51(3.76,5.28) |
| **Botswana** | 0(0 to 0) | 0.07(0.02 to 0.17) | 1(0 to 2) | 0.14(0.05 to 0.28) | 2.06(0.46,3.68) | 7(2 to 16) | 3.30(1.02 to 7.77) | 42(16 to 84) | 6.60(2.55 to 13.08) | 2.09(0.50,3.71) |
| **Brazil** | 43(14 to 88) | 0.14(0.04 to 0.28) | 83(41 to 132) | 0.13(0.06 to 0.20) | -0.28(-0.77,0.20) | 2093(678 to 4237) | 6.60(2.14 to 13.36) | 3925(1934 to 6234) | 5.98(2.95 to 9.51) | -0.30(-0.80,0.19) |
| **Brunei Darussalam** | 0(0 to 0) | 0.17(0.01 to 0.46) | 0(0 to 0) | 0.12(0.02 to 0.28) | -1.08(-1.97,-0.18) | 4(0 to 11) | 7.95(0.50 to 22.32) | 8(2 to 18) | 5.76(1.12 to 13.08) | -1.12(-1.97,-0.26) |
| **Bulgaria** | 8(4 to 14) | 0.34(0.15 to 0.60) | 9(5 to 14) | 0.43(0.25 to 0.67) | 0.56(-0.85,1.98) | 386(168 to 682) | 16.16(7.02 to 28.52) | 418(238 to 643) | 20.30(11.55 to 31.29) | 0.48(-0.93,1.92) |
| **Burkina Faso** | 0(0 to 1) | 0.02(0.01 to 0.04) | 1(0 to 2) | 0.02(0.01 to 0.04) | 0.25(-0.67,1.17) | 14(5 to 28) | 0.85(0.34 to 1.73) | 38(14 to 82) | 0.93(0.35 to 2.00) | 0.35(-0.87,1.59) |
| **Burundi** | 0(0 to 0) | 0.02(0.01 to 0.04) | 0(0 to 1) | 0.01(0.00 to 0.03) | -1.66(-2.02,-1.30) | 8(3 to 16) | 0.87(0.35 to 1.84) | 11(4 to 26) | 0.53(0.19 to 1.21) | -1.64(-2.08,-1.20) |
| **Cabo Verde** | 0(0 to 0) | 0.08(0.04 to 0.16) | 0(0 to 0) | 0.19(0.08 to 0.36) | 2.52(1.84,3.20) | 3(1 to 5) | 4.29(1.98 to 7.97) | 13(5 to 25) | 9.14(3.89 to 17.64) | 2.55(1.94,3.17) |
| **Cambodia** | 1(0 to 2) | 0.06(0.02 to 0.13) | 4(2 to 9) | 0.09(0.04 to 0.21) | 1.84(1.62,2.06) | 53(17 to 122) | 2.81(0.89 to 6.40) | 187(75 to 415) | 4.52(1.81 to 9.97) | 1.83(1.62,2.05) |
| **Cameroon** | 1(0 to 1) | 0.04(0.02 to 0.07) | 2(1 to 5) | 0.04(0.02 to 0.09) | 0.24(-0.21,0.69) | 31(13 to 61) | 1.78(0.73 to 3.49) | 119(45 to 264) | 1.96(0.74 to 4.33) | 0.28(-0.16,0.73) |
| **Canada** | 24(6 to 47) | 0.31(0.08 to 0.62) | 4(1 to 8) | 0.04(0.01 to 0.08) | -6.61(-7.19,-6.02) | 1112(290 to 2198) | 14.60(3.80 to 28.83) | 180(58 to 358) | 1.78(0.58 to 3.55) | -6.57(-7.16,-5.97) |
| **Central African Republic** | 0(0 to 0) | 0.02(0.01 to 0.05) | 0(0 to 0) | 0.02(0.01 to 0.04) | -0.37(-0.76,0.02) | 5(2 to 10) | 1.09(0.46 to 2.20) | 10(5 to 21) | 0.99(0.43 to 1.94) | -0.35(-0.75,0.05) |
| **Chad** | 0(0 to 0) | 0.01(0.01 to 0.03) | 1(0 to 1) | 0.02(0.01 to 0.05) | 1.29(0.77,1.82) | 7(3 to 14) | 0.71(0.29 to 1.39) | 29(11 to 60) | 1.06(0.41 to 2.21) | 1.33(0.80,1.86) |
| **Chile** | 4(1 to 9) | 0.14(0.04 to 0.29) | 9(5 to 15) | 0.18(0.09 to 0.28) | 0.78(0.29,1.27) | 202(64 to 426) | 6.47(2.05 to 13.64) | 442(214 to 710) | 8.44(4.09 to 13.56) | 0.83(0.36,1.31) |
| **China** | 745(330 to 1447) | 0.29(0.13 to 0.57) | 2888(1479 to 4448) | 0.69(0.35 to 1.07) | 2.78(2.54,3.01) | 36417(16140 to 70743) | 13.99(6.21 to 27.20) | 136710(69994 to 210449) | 33.22(17.00 to 51.11) | 2.77(2.54,3.00) |
| **Colombia** | 15(5 to 26) | 0.22(0.08 to 0.40) | 14(8 to 23) | 0.11(0.06 to 0.17) | -2.54(-2.94,-2.13) | 705(258 to 1275) | 10.63(3.90 to 19.19) | 692(360 to 1102) | 5.19(2.70 to 8.26) | -2.52(-2.93,-2.10) |
| **Comoros** | 0(0 to 0) | 0.01(0.01 to 0.03) | 0(0 to 0) | 0.01(0.00 to 0.03) | 0.17(-1.19,1.56) | 0(0 to 1) | 0.60(0.24 to 1.29) | 1(0 to 2) | 0.62(0.23 to 1.34) | 0.20(-1.20,1.63) |
| **Congo** | 0(0 to 1) | 0.07(0.02 to 0.16) | 2(1 to 4) | 0.14(0.04 to 0.33) | 2.35(1.52,3.18) | 12(3 to 28) | 3.15(0.85 to 7.33) | 82(25 to 196) | 6.44(1.93 to 15.41) | 2.38(1.53,3.24) |
| **Cook Islands** | 0(0 to 0) | 0.05(0.00 to 0.15) | 0(0 to 0) | 0.04(0.00 to 0.09) | -1.30(-1.80,-0.80) | 0(0 to 0) | 2.54(0.18 to 7.24) | 0(0 to 0) | 1.65(0.00 to 4.45) | -1.32(-1.82,-0.82) |
| **Costa Rica** | 1(0 to 1) | 0.11(0.04 to 0.21) | 1(1 to 2) | 0.07(0.04 to 0.12) | -1.28(-2.40,-0.15) | 30(11 to 58) | 5.12(1.82 to 9.89) | 48(26 to 79) | 3.52(1.92 to 5.84) | -1.24(-2.34,-0.12) |
| **Coted'Ivoire** | 0(0 to 1) | 0.02(0.01 to 0.04) | 1(0 to 3) | 0.02(0.01 to 0.05) | 0.95(-0.10,2.01) | 17(7 to 33) | 0.92(0.39 to 1.80) | 62(23 to 131) | 1.19(0.45 to 2.50) | 0.97(-0.08,2.04) |
| **Croatia** | 5(2 to 8) | 0.35(0.16 to 0.60) | 3(2 to 5) | 0.25(0.15 to 0.39) | -1.20(-3.48,1.14) | 214(96 to 368) | 16.29(7.32 to 28.05) | 140(80 to 214) | 11.79(6.75 to 18.03) | -1.23(-3.50,1.10) |
| **Cuba** | 12(3 to 25) | 0.44(0.12 to 0.93) | 10(4 to 17) | 0.30(0.12 to 0.52) | -1.11(-2.27,0.07) | 562(151 to 1195) | 20.67(5.57 to 43.95) | 447(181 to 786) | 14.07(5.70 to 24.71) | -1.13(-2.26,0.01) |
| **Cyprus** | 0(0 to 0) | 0.12(0.05 to 0.23) | 0(0 to 1) | 0.10(0.05 to 0.16) | -0.86(-1.66,-0.06) | 11(4 to 22) | 5.63(2.12 to 10.67) | 20(10 to 35) | 4.48(2.19 to 7.60) | -0.87(-1.50,-0.23) |
| **Czechia** | 13(6 to 21) | 0.42(0.20 to 0.69) | 6(3 to 9) | 0.17(0.10 to 0.26) | -2.98(-4.40,-1.54) | 590(286 to 976) | 19.37(9.37 to 32.03) | 274(160 to 413) | 8.24(4.82 to 12.47) | -2.88(-4.27,-1.47) |
| **Democratic People's Republic of Korea** | 11(4 to 26) | 0.21(0.07 to 0.48) | 10(4 to 21) | 0.13(0.05 to 0.27) | -1.41(-1.57,-1.25) | 525(169 to 1215) | 9.94(3.19 to 22.93) | 481(184 to 981) | 6.38(2.45 to 13.02) | -1.41(-1.59,-1.24) |
| **Democratic Republic of the Congo** | 1(0 to 2) | 0.02(0.01 to 0.04) | 3(1 to 5) | 0.02(0.01 to 0.03) | -0.45(-0.76,-0.14) | 54(23 to 109) | 0.88(0.38 to 1.77) | 124(51 to 252) | 0.78(0.32 to 1.59) | -0.46(-0.77,-0.15) |
| **Denmark** | 11(4 to 19) | 0.69(0.28 to 1.24) | 2(1 to 4) | 0.14(0.07 to 0.23) | -5.30(-5.90,-4.70) | 485(195 to 873) | 31.75(12.73 to 57.17) | 101(50 to 166) | 6.61(3.29 to 10.93) | -5.20(-5.79,-4.61) |
| **Djibouti** | 0(0 to 0) | 0.07(0.02 to 0.14) | 0(0 to 1) | 0.11(0.04 to 0.26) | 1.61(0.88,2.34) | 2(1 to 5) | 3.24(1.14 to 6.75) | 16(6 to 37) | 5.34(1.91 to 12.15) | 1.64(0.88,2.41) |
| **Dominica** | 0(0 to 0) | 0.10(0.02 to 0.24) | 0(0 to 0) | 0.20(0.06 to 0.40) | 2.23(2.03,2.44) | 1(0 to 1) | 4.59(1.04 to 11.18) | 2(1 to 3) | 9.22(3.06 to 18.69) | 2.27(2.06,2.47) |
| **Dominican Republic** | 1(0 to 3) | 0.10(0.02 to 0.24) | 7(2 to 15) | 0.26(0.08 to 0.55) | 3.24(2.46,4.02) | 62(14 to 155) | 4.63(1.04 to 11.58) | 344(101 to 731) | 12.39(3.65 to 26.32) | 3.23(2.48,3.99) |
| **Ecuador** | 4(2 to 7) | 0.22(0.09 to 0.38) | 6(3 to 11) | 0.13(0.06 to 0.24) | -1.94(-3.38,-0.48) | 208(82 to 364) | 10.65(4.22 to 18.68) | 286(136 to 518) | 6.25(2.97 to 11.33) | -1.97(-3.39,-0.53) |
| **Egypt** | 21(11 to 36) | 0.20(0.10 to 0.33) | 108(59 to 174) | 0.46(0.25 to 0.74) | 2.64(1.89,3.40) | 1064(555 to 1784) | 9.68(5.06 to 16.19) | 5309(2901 to 8598) | 22.25(12.17 to 36.05) | 2.64(1.89,3.40) |
| **El Salvador** | 1(0 to 2) | 0.10(0.04 to 0.20) | 3(1 to 5) | 0.15(0.06 to 0.29) | 1.43(0.36,2.52) | 45(17 to 93) | 4.68(1.72 to 9.62) | 125(51 to 238) | 7.15(2.91 to 13.64) | 1.45(0.37,2.54) |
| **Equatorial Guinea** | 0(0 to 0) | 0.03(0.01 to 0.07) | 1(0 to 1) | 0.26(0.09 to 0.55) | 7.26(6.48,8.05) | 1(0 to 2) | 1.45(0.55 to 3.10) | 34(12 to 73) | 12.41(4.18 to 26.15) | 7.29(6.51,8.08) |
| **Eritrea** | 0(0 to 0) | 0.03(0.01 to 0.06) | 1(0 to 1) | 0.04(0.02 to 0.09) | 0.94(0.35,1.54) | 9(3 to 17) | 1.49(0.59 to 3.01) | 27(11 to 60) | 2.03(0.79 to 4.44) | 0.98(0.38,1.58) |
| **Estonia** | 1(0 to 1) | 0.17(0.06 to 0.34) | 0(0 to 0) | 0.03(0.01 to 0.06) | -6.17(-6.78,-5.54) | 34(12 to 68) | 8.07(2.91 to 16.06) | 5(1 to 10) | 1.39(0.41 to 2.74) | -6.15(-6.78,-5.52) |
| **Eswatini** | 0(0 to 0) | 0.07(0.02 to 0.18) | 0(0 to 1) | 0.18(0.05 to 0.46) | 2.92(2.44,3.41) | 4(1 to 11) | 3.35(0.98 to 8.24) | 21(5 to 52) | 8.57(2.21 to 21.52) | 2.94(2.45,3.42) |
| **Ethiopia** | 1(0 to 2) | 0.01(0.01 to 0.03) | 4(2 to 6) | 0.02(0.01 to 0.03) | 1.09(0.64,1.55) | 53(21 to 102) | 0.66(0.26 to 1.27) | 182(89 to 308) | 0.90(0.44 to 1.53) | 1.04(0.69,1.39) |
| **Fiji** | 0(0 to 0) | 0.03(0.01 to 0.10) | 0(0 to 0) | 0.07(0.02 to 0.17) | 2.57(1.62,3.53) | 2(0 to 8) | 1.56(0.27 to 4.86) | 8(2 to 18) | 3.31(0.81 to 7.83) | 2.58(1.64,3.53) |
| **Finland** | 1(0 to 3) | 0.08(0.01 to 0.19) | 0(0 to 1) | 0.02(0.00 to 0.05) | -4.48(-5.41,-3.54) | 59(9 to 134) | 3.88(0.59 to 8.83) | 14(2 to 34) | 1.06(0.14 to 2.53) | -4.44(-5.36,-3.50) |
| **France** | 32(13 to 57) | 0.22(0.09 to 0.38) | 28(15 to 46) | 0.16(0.08 to 0.25) | -1.22(-1.48,-0.95) | 1532(602 to 2703) | 10.34(4.06 to 18.25) | 1306(705 to 2105) | 7.31(3.94 to 11.78) | -1.29(-1.56,-1.02) |
| **Gabon** | 0(0 to 1) | 0.17(0.06 to 0.39) | 1(0 to 2) | 0.21(0.09 to 0.42) | 0.75(0.24,1.26) | 12(4 to 27) | 8.07(2.70 to 18.22) | 40(16 to 80) | 10.09(4.04 to 19.99) | 0.75(0.24,1.26) |
| **Gambia** | 0(0 to 0) | 0.01(0.00 to 0.02) | 0(0 to 0) | 0.01(0.00 to 0.03) | 1.09(0.03,2.17) | 1(0 to 1) | 0.42(0.18 to 0.82) | 2(1 to 5) | 0.57(0.20 to 1.22) | 1.11(0.04,2.18) |
| **Georgia** | 2(1 to 5) | 0.19(0.06 to 0.38) | 1(1 to 2) | 0.12(0.06 to 0.20) | -1.30(-3.15,0.59) | 122(38 to 242) | 9.15(2.87 to 18.19) | 58(26 to 94) | 5.93(2.63 to 9.69) | -1.06(-2.51,0.42) |
| **Germany** | 88(39 to 151) | 0.41(0.18 to 0.71) | 27(15 to 42) | 0.13(0.07 to 0.20) | -3.82(-4.32,-3.31) | 4099(1800 to 7029) | 19.24(8.45 to 32.99) | 1248(686 to 1940) | 5.87(3.23 to 9.13) | -3.80(-4.30,-3.30) |
| **Ghana** | 1(0 to 1) | 0.02(0.01 to 0.04) | 3(1 to 6) | 0.04(0.01 to 0.07) | 2.01(1.29,2.74) | 26(11 to 50) | 0.99(0.43 to 1.88) | 134(52 to 275) | 1.74(0.68 to 3.55) | 2.00(1.28,2.73) |
| **Greece** | 9(4 to 15) | 0.33(0.15 to 0.55) | 6(4 to 9) | 0.20(0.12 to 0.30) | -1.45(-2.21,-0.69) | 423(195 to 705) | 15.59(7.20 to 25.97) | 298(174 to 434) | 9.59(5.62 to 13.97) | -1.47(-2.23,-0.70) |
| **Greenland** | 0(0 to 0) | 0.42(0.00 to 1.25) | 0(0 to 0) | 0.18(0.01 to 0.54) | -2.49(-2.96,-2.02) | 2(0 to 7) | 19.84(0.08 to 58.72) | 1(0 to 3) | 8.46(0.25 to 25.11) | -2.55(-3.02,-2.08) |
| **Grenada** | 0(0 to 0) | 0.14(0.03 to 0.32) | 0(0 to 0) | 0.18(0.07 to 0.34) | 0.88(0.41,1.36) | 1(0 to 2) | 6.54(1.54 to 15.51) | 2(1 to 4) | 8.37(3.19 to 16.03) | 0.82(0.35,1.30) |
| **Guam** | 0(0 to 0) | 0.09(0.00 to 0.25) | 0(0 to 0) | 0.20(0.08 to 0.35) | 2.71(1.02,4.43) | 1(0 to 3) | 4.32(0.00 to 11.90) | 4(2 to 7) | 9.27(3.97 to 16.39) | 2.58(1.21,3.95) |
| **Guatemala** | 2(1 to 4) | 0.16(0.06 to 0.30) | 3(1 to 6) | 0.09(0.03 to 0.18) | -1.74(-3.68,0.25) | 108(42 to 206) | 7.79(3.04 to 14.72) | 169(61 to 314) | 4.52(1.63 to 8.41) | -1.79(-3.78,0.25) |
| **Guinea** | 0(0 to 0) | 0.02(0.01 to 0.04) | 1(0 to 1) | 0.02(0.01 to 0.05) | 0.35(-0.24,0.95) | 11(5 to 20) | 0.99(0.44 to 1.87) | 27(10 to 59) | 1.09(0.40 to 2.41) | 0.38(-0.23,0.99) |
| **Guinea-Bissau** | 0(0 to 0) | 0.04(0.02 to 0.07) | 0(0 to 0) | 0.04(0.02 to 0.08) | 0.08(-0.27,0.44) | 3(1 to 6) | 1.76(0.76 to 3.43) | 7(3 to 15) | 1.81(0.74 to 3.73) | 0.11(-0.25,0.47) |
| **Guyana** | 0(0 to 0) | 0.06(0.01 to 0.15) | 0(0 to 1) | 0.17(0.06 to 0.34) | 3.64(2.72,4.57) | 4(1 to 10) | 2.72(0.57 to 6.97) | 16(6 to 31) | 8.22(2.88 to 16.29) | 3.66(2.69,4.64) |
| **Haiti** | 0(0 to 1) | 0.04(0.01 to 0.09) | 1(0 to 2) | 0.03(0.01 to 0.08) | -0.08(-0.39,0.23) | 20(5 to 51) | 1.71(0.43 to 4.38) | 51(16 to 119) | 1.60(0.52 to 3.73) | -0.08(-0.39,0.24) |
| **Honduras** | 1(0 to 2) | 0.15(0.06 to 0.32) | 4(1 to 9) | 0.17(0.05 to 0.38) | 0.54(0.19,0.89) | 57(22 to 120) | 7.40(2.82 to 15.49) | 190(60 to 429) | 7.98(2.53 to 17.93) | 0.45(0.10,0.79) |
| **Hungary** | 25(10 to 44) | 0.85(0.34 to 1.46) | 10(6 to 15) | 0.31(0.18 to 0.47) | -3.63(-4.56,-2.69) | 1199(484 to 2074) | 40.16(16.22 to 69.49) | 453(261 to 689) | 14.59(8.39 to 22.26) | -3.59(-4.12,-3.06) |
| **Iceland** | 0(0 to 0) | 0.16(0.01 to 0.40) | 0(0 to 0) | 0.07(0.01 to 0.17) | -2.95(-3.76,-2.13) | 5(0 to 11) | 7.58(0.58 to 18.76) | 3(1 to 8) | 3.49(0.59 to 8.25) | -2.87(-3.65,-2.08) |
| **India** | 62(32 to 107) | 0.04(0.02 to 0.06) | 483(273 to 736) | 0.14(0.08 to 0.21) | 4.26(3.61,4.91) | 3040(1542 to 5204) | 1.81(0.92 to 3.09) | 23158(13080 to 35373) | 6.46(3.65 to 9.86) | 4.23(3.57,4.90) |
| **Indonesia** | 53(18 to 112) | 0.14(0.05 to 0.30) | 214(89 to 378) | 0.26(0.11 to 0.46) | 1.97(1.56,2.38) | 2580(861 to 5484) | 6.77(2.26 to 14.36) | 10187(4244 to 18033) | 12.52(5.23 to 22.17) | 1.95(1.54,2.36) |
| **Iran (Islamic Republic of)** | 19(11 to 30) | 0.21(0.13 to 0.33) | 66(40 to 95) | 0.25(0.15 to 0.35) | 0.40(0.04,0.76) | 968(574 to 1501) | 10.47(6.19 to 16.22) | 3267(1986 to 4667) | 12.20(7.42 to 17.45) | 0.40(0.03,0.78) |
| **Iraq** | 7(3 to 12) | 0.24(0.11 to 0.44) | 30(15 to 52) | 0.32(0.16 to 0.56) | 0.92(0.54,1.29) | 325(144 to 583) | 11.58(5.15 to 20.73) | 1442(715 to 2507) | 15.29(7.60 to 26.55) | 0.86(0.51,1.22) |
| **Ireland** | 2(1 to 4) | 0.28(0.10 to 0.54) | 2(1 to 3) | 0.10(0.04 to 0.17) | -3.41(-4.02,-2.80) | 111(38 to 210) | 13.23(4.52 to 25.03) | 71(30 to 123) | 4.69(2.00 to 8.18) | -3.35(-3.94,-2.75) |
| **Israel** | 3(1 to 5) | 0.27(0.13 to 0.45) | 4(2 to 6) | 0.16(0.09 to 0.23) | -1.77(-2.94,-0.59) | 138(67 to 232) | 12.50(6.03 to 21.00) | 182(109 to 266) | 7.50(4.47 to 10.97) | -1.78(-2.36,-1.20) |
| **Italy** | 56(29 to 87) | 0.36(0.19 to 0.57) | 27(17 to 39) | 0.15(0.09 to 0.21) | -2.98(-3.34,-2.62) | 2631(1357 to 4101) | 17.21(8.87 to 26.82) | 1248(762 to 1806) | 7.00(4.26 to 10.12) | -2.99(-3.36,-2.63) |
| **Jamaica** | 0(0 to 1) | 0.07(0.02 to 0.15) | 1(0 to 2) | 0.16(0.06 to 0.30) | 2.80(2.28,3.33) | 12(4 to 29) | 3.04(0.86 to 7.12) | 57(22 to 106) | 7.49(2.96 to 14.04) | 2.89(2.38,3.40) |
| **Japan** | 60(11 to 129) | 0.15(0.03 to 0.33) | 29(14 to 48) | 0.08(0.04 to 0.13) | -2.20(-2.76,-1.64) | 2847(534 to 6101) | 7.35(1.38 to 15.77) | 1351(664 to 2234) | 3.83(1.88 to 6.33) | -2.18(-2.75,-1.61) |
| **Jordan** | 1(0 to 1) | 0.15(0.08 to 0.27) | 4(2 to 8) | 0.16(0.08 to 0.27) | -0.06(-0.86,0.75) | 39(19 to 68) | 7.24(3.58 to 12.56) | 209(101 to 363) | 7.50(3.62 to 13.00) | -0.04(-0.85,0.78) |
| **Kazakhstan** | 11(4 to 20) | 0.31(0.10 to 0.58) | 9(5 to 13) | 0.17(0.10 to 0.25) | -1.90(-2.63,-1.16) | 529(179 to 999) | 14.75(4.99 to 27.84) | 428(244 to 643) | 8.10(4.62 to 12.18) | -1.91(-2.63,-1.18) |
| **Kenya** | 0(0 to 0) | 0.01(0.00 to 0.01) | 2(1 to 3) | 0.02(0.01 to 0.04) | 4.49(3.51,5.47) | 8(3 to 18) | 0.25(0.09 to 0.54) | 88(38 to 168) | 0.89(0.39 to 1.70) | 4.47(3.49,5.46) |
| **Kiribati** | 0(0 to 0) | 0.01(0.00 to 0.03) | 0(0 to 0) | 0.02(0.00 to 0.05) | 1.57(1.38,1.76) | 0(0 to 0) | 0.50(0.12 to 1.44) | 0(0 to 1) | 0.81(0.22 to 2.19) | 1.57(1.43,1.70) |
| **Kuwait** | 1(0 to 1) | 0.25(0.15 to 0.37) | 3(2 to 4) | 0.15(0.09 to 0.22) | -1.86(-3.73,0.04) | 33(20 to 48) | 11.67(7.16 to 17.06) | 129(76 to 189) | 7.22(4.24 to 10.59) | -1.75(-3.71,0.24) |
| **Kyrgyzstan** | 1(0 to 3) | 0.18(0.05 to 0.42) | 2(1 to 3) | 0.09(0.04 to 0.17) | -2.38(-3.10,-1.65) | 68(20 to 157) | 8.80(2.65 to 20.31) | 73(30 to 135) | 4.41(1.83 to 8.16) | -2.33(-3.02,-1.63) |
| **Lao People's Democratic Republic** | 0(0 to 1) | 0.07(0.02 to 0.17) | 3(1 to 6) | 0.16(0.05 to 0.36) | 2.90(2.76,3.04) | 23(7 to 59) | 3.12(0.94 to 8.06) | 133(39 to 309) | 7.47(2.18 to 17.37) | 2.94(2.81,3.08) |
| **Latvia** | 2(1 to 4) | 0.27(0.11 to 0.48) | 1(0 to 1) | 0.09(0.05 to 0.15) | -3.43(-4.73,-2.11) | 93(39 to 167) | 12.65(5.28 to 22.64) | 23(12 to 37) | 4.45(2.34 to 7.07) | -3.42(-4.73,-2.09) |
| **Lebanon** | 1(1 to 3) | 0.21(0.09 to 0.40) | 4(2 to 8) | 0.27(0.12 to 0.48) | 0.51(0.21,0.82) | 65(27 to 124) | 10.00(4.20 to 18.93) | 199(88 to 362) | 12.48(5.56 to 22.65) | 0.50(0.20,0.80) |
| **Lesotho** | 0(0 to 0) | 0.02(0.01 to 0.04) | 0(0 to 0) | 0.05(0.02 to 0.12) | 3.54(2.80,4.28) | 3(1 to 6) | 0.86(0.36 to 1.76) | 10(3 to 21) | 2.54(0.84 to 5.56) | 3.55(2.81,4.30) |
| **Liberia** | 0(0 to 0) | 0.02(0.01 to 0.03) | 0(0 to 0) | 0.02(0.01 to 0.03) | 0.11(-0.96,1.20) | 3(1 to 5) | 0.74(0.33 to 1.40) | 9(4 to 19) | 0.79(0.31 to 1.59) | 0.17(-0.88,1.23) |
| **Libya** | 1(0 to 2) | 0.15(0.07 to 0.28) | 5(2 to 10) | 0.23(0.10 to 0.43) | 1.13(0.80,1.46) | 42(18 to 77) | 7.30(3.21 to 13.37) | 238(107 to 450) | 10.76(4.82 to 20.33) | 1.12(0.77,1.46) |
| **Lithuania** | 2(1 to 4) | 0.23(0.09 to 0.41) | 1(0 to 1) | 0.08(0.04 to 0.12) | -4.07(-5.51,-2.60) | 105(43 to 187) | 10.76(4.40 to 19.13) | 27(13 to 43) | 3.60(1.79 to 5.75) | -4.05(-5.48,-2.60) |
| **Luxembourg** | 0(0 to 0) | 0.27(0.10 to 0.48) | 0(0 to 0) | 0.08(0.04 to 0.14) | -3.76(-4.97,-2.54) | 13(5 to 24) | 12.52(4.93 to 22.46) | 8(4 to 13) | 3.87(1.90 to 6.45) | -3.79(-4.96,-2.60) |
| **Madagascar** | 0(0 to 0) | 0.01(0.00 to 0.01) | 1(0 to 1) | 0.01(0.00 to 0.02) | 0.69(0.39,0.98) | 7(3 to 13) | 0.38(0.18 to 0.71) | 26(11 to 48) | 0.47(0.21 to 0.86) | 0.71(0.41,1.01) |
| **Malawi** | 0(0 to 0) | 0.00(0.00 to 0.00) | 0(0 to 0) | 0.00(0.00 to 0.01) | 0.81(0.36,1.25) | 2(1 to 4) | 0.12(0.06 to 0.24) | 5(2 to 11) | 0.15(0.06 to 0.32) | 0.80(0.35,1.25) |
| **Malaysia** | 7(3 to 14) | 0.21(0.08 to 0.42) | 16(8 to 29) | 0.21(0.10 to 0.37) | 0.20(-0.58,0.98) | 344(124 to 682) | 9.98(3.57 to 19.79) | 778(389 to 1378) | 9.81(4.90 to 17.36) | 0.18(-0.60,0.96) |
| **Maldives** | 0(0 to 0) | 0.06(0.01 to 0.18) | 0(0 to 0) | 0.05(0.02 to 0.09) | -1.05(-1.76,-0.35) | 1(0 to 3) | 2.97(0.63 to 8.49) | 3(1 to 5) | 2.26(0.94 to 4.27) | -1.01(-1.69,-0.31) |
| **Mali** | 0(0 to 1) | 0.03(0.01 to 0.05) | 1(0 to 2) | 0.03(0.01 to 0.06) | 0.58(0.43,0.74) | 18(9 to 34) | 1.27(0.59 to 2.34) | 57(23 to 114) | 1.53(0.63 to 3.08) | 0.60(0.44,0.76) |
| **Malta** | 0(0 to 0) | 0.11(0.04 to 0.20) | 0(0 to 0) | 0.12(0.06 to 0.18) | -0.11(-1.25,1.05) | 6(2 to 10) | 5.20(2.06 to 9.55) | 7(4 to 10) | 5.39(2.98 to 8.41) | -0.09(-1.22,1.05) |
| **Marshall Islands** | 0(0 to 0) | 0.04(0.01 to 0.13) | 0(0 to 0) | 0.08(0.02 to 0.23) | 1.88(1.60,2.16) | 0(0 to 0) | 2.08(0.42 to 6.18) | 1(0 to 2) | 3.71(0.79 to 10.91) | 1.90(1.64,2.15) |
| **Mauritania** | 0(0 to 0) | 0.05(0.02 to 0.10) | 1(0 to 1) | 0.08(0.03 to 0.17) | 1.46(0.90,2.02) | 8(3 to 16) | 2.39(0.98 to 4.81) | 32(13 to 66) | 3.88(1.57 to 8.00) | 1.50(0.93,2.06) |
| **Mauritius** | 0(0 to 0) | 0.06(0.02 to 0.11) | 0(0 to 0) | 0.06(0.02 to 0.11) | -0.04(-1.91,1.87) | 7(3 to 13) | 2.68(1.05 to 5.12) | 10(3 to 19) | 2.82(0.89 to 5.28) | -0.03(-1.84,1.82) |
| **Mexico** | 36(17 to 56) | 0.22(0.11 to 0.35) | 30(17 to 46) | 0.08(0.04 to 0.12) | -3.23(-4.18,-2.27) | 1751(854 to 2734) | 10.78(5.25 to 16.82) | 1420(788 to 2199) | 3.84(2.13 to 5.95) | -3.25(-4.20,-2.29) |
| **Micronesia (Federated States of)** | 0(0 to 0) | 0.05(0.01 to 0.15) | 0(0 to 0) | 0.09(0.02 to 0.26) | 1.94(1.80,2.08) | 0(0 to 1) | 2.38(0.50 to 7.21) | 1(0 to 3) | 4.32(1.03 to 12.16) | 1.95(1.82,2.07) |
| **Monaco** | 0(0 to 0) | 0.25(0.03 to 0.57) | 0(0 to 0) | 0.44(0.18 to 0.82) | 1.87(1.26,2.48) | 1(0 to 3) | 12.02(1.65 to 26.96) | 2(1 to 4) | 21.08(8.50 to 39.14) | 1.89(1.28,2.51) |
| **Mongolia** | 0(0 to 1) | 0.11(0.04 to 0.25) | 3(1 to 5) | 0.29(0.11 to 0.49) | 2.94(2.00,3.89) | 19(7 to 42) | 5.49(1.92 to 11.93) | 129(50 to 218) | 13.96(5.37 to 23.59) | 2.94(2.02,3.88) |
| **Montenegro** | 1(0 to 2) | 0.68(0.25 to 1.20) | 1(0 to 1) | 0.46(0.21 to 0.74) | -1.15(-2.15,-0.14) | 47(17 to 84) | 31.71(11.60 to 56.48) | 39(18 to 62) | 21.61(9.96 to 34.56) | -1.14(-2.07,-0.21) |
| **Morocco** | 1(0 to 2) | 0.02(0.01 to 0.04) | 5(2 to 9) | 0.05(0.02 to 0.09) | 3.20(2.92,3.47) | 42(19 to 79) | 0.88(0.40 to 1.68) | 231(107 to 444) | 2.30(1.07 to 4.42) | 3.18(2.91,3.45) |
| **Mozambique** | 0(0 to 0) | 0.01(0.00 to 0.01) | 0(0 to 1) | 0.01(0.00 to 0.02) | 1.47(1.04,1.90) | 6(3 to 10) | 0.24(0.12 to 0.42) | 19(8 to 39) | 0.37(0.15 to 0.74) | 1.47(1.09,1.86) |
| **Myanmar** | 8(3 to 19) | 0.11(0.04 to 0.25) | 29(11 to 59) | 0.19(0.07 to 0.39) | 1.93(1.73,2.12) | 414(137 to 950) | 5.23(1.74 to 11.93) | 1397(504 to 2857) | 9.20(3.31 to 18.82) | 1.93(1.73,2.12) |
| **Namibia** | 0(0 to 0) | 0.03(0.01 to 0.07) | 0(0 to 1) | 0.07(0.02 to 0.15) | 2.40(1.81,3.01) | 4(1 to 8) | 1.56(0.51 to 3.39) | 18(6 to 39) | 3.18(1.02 to 6.96) | 2.41(1.81,3.01) |
| **Nauru** | 0(0 to 0) | 0.09(0.00 to 0.41) | 0(0 to 0) | 0.11(0.01 to 0.31) | 0.44(0.12,0.76) | 0(0 to 0) | 4.26(0.00 to 19.27) | 0(0 to 0) | 4.98(0.60 to 14.51) | 0.45(0.13,0.78) |
| **Nepal** | 1(0 to 2) | 0.03(0.01 to 0.06) | 5(2 to 10) | 0.06(0.03 to 0.12) | 2.15(1.32,2.98) | 50(17 to 110) | 1.40(0.49 to 3.09) | 242(104 to 469) | 3.06(1.32 to 5.90) | 2.15(1.32,3.00) |
| **Netherlands** | 21(9 to 36) | 0.52(0.23 to 0.88) | 8(5 to 13) | 0.18(0.10 to 0.28) | -3.43(-4.10,-2.76) | 1001(442 to 1701) | 24.12(10.65 to 41.00) | 387(214 to 597) | 8.67(4.78 to 13.40) | -3.42(-4.08,-2.76) |
| **New Zealand** | 1(0 to 3) | 0.12(0.00 to 0.36) | 1(0 to 2) | 0.09(0.03 to 0.16) | -1.06(-1.43,-0.69) | 47(1 to 145) | 5.39(0.14 to 16.52) | 54(19 to 100) | 4.01(1.39 to 7.43) | -1.04(-1.41,-0.67) |
| **Nicaragua** | 0(0 to 0) | 0.04(0.01 to 0.08) | 1(0 to 1) | 0.03(0.01 to 0.07) | -0.51(-1.20,0.18) | 12(4 to 25) | 1.86(0.69 to 3.95) | 27(9 to 57) | 1.56(0.56 to 3.35) | -0.54(-1.23,0.16) |
| **Niger** | 0(0 to 1) | 0.02(0.01 to 0.04) | 1(0 to 1) | 0.01(0.00 to 0.04) | -0.60(-1.89,0.70) | 11(4 to 25) | 0.88(0.31 to 1.99) | 25(8 to 61) | 0.70(0.24 to 1.70) | -0.58(-1.85,0.72) |
| **Nigeria** | 1(0 to 2) | 0.01(0.00 to 0.02) | 6(2 to 12) | 0.01(0.01 to 0.03) | 1.90(1.13,2.67) | 48(18 to 97) | 0.36(0.14 to 0.72) | 279(101 to 569) | 0.66(0.24 to 1.34) | 1.92(1.14,2.70) |
| **Niue** | 0(0 to 0) | 0.06(0.02 to 0.16) | 0(0 to 0) | 0.07(0.01 to 0.17) | 0.48(0.17,0.79) | 0(0 to 0) | 2.93(0.73 to 7.39) | 0(0 to 0) | 3.51(0.48 to 8.48) | 0.59(0.27,0.92) |
| **North Macedonia** | 2(1 to 3) | 0.36(0.13 to 0.63) | 2(1 to 4) | 0.34(0.18 to 0.55) | -0.23(-0.88,0.42) | 86(32 to 150) | 16.96(6.34 to 29.69) | 108(56 to 173) | 16.05(8.34 to 25.81) | -0.26(-0.90,0.38) |
| **Northern Mariana Islands** | 0(0 to 0) | 0.15(0.00 to 0.42) | 0(0 to 0) | 0.14(0.06 to 0.25) | -0.51(-1.27,0.26) | 1(0 to 2) | 6.98(0.00 to 19.94) | 1(0 to 2) | 6.47(2.66 to 12.09) | -0.42(-0.99,0.16) |
| **Norway** | 2(0 to 3) | 0.16(0.04 to 0.30) | 1(0 to 1) | 0.05(0.02 to 0.09) | -4.00(-4.81,-3.18) | 83(23 to 159) | 7.30(2.02 to 14.02) | 31(11 to 60) | 2.13(0.76 to 4.08) | -4.00(-4.81,-3.18) |
| **Oman** | 0(0 to 0) | 0.06(0.03 to 0.13) | 1(0 to 1) | 0.05(0.03 to 0.10) | -0.53(-1.10,0.04) | 7(3 to 14) | 3.01(1.26 to 5.94) | 26(12 to 48) | 2.60(1.26 to 4.87) | -0.48(-1.03,0.08) |
| **Pakistan** | 8(3 to 15) | 0.04(0.02 to 0.08) | 55(23 to 107) | 0.11(0.05 to 0.21) | 2.92(1.99,3.85) | 362(156 to 712) | 1.95(0.84 to 3.82) | 2683(1132 to 5199) | 5.16(2.18 to 10.00) | 2.94(2.02,3.88) |
| **Palau** | 0(0 to 0) | 0.18(0.00 to 0.55) | 0(0 to 0) | 0.17(0.01 to 0.39) | -0.33(-1.29,0.63) | 0(0 to 1) | 8.53(0.04 to 26.08) | 0(0 to 1) | 8.13(0.71 to 18.95) | -0.30(-1.25,0.67) |
| **Palestine** | 0(0 to 1) | 0.13(0.06 to 0.27) | 2(1 to 4) | 0.20(0.11 to 0.35) | 1.13(0.44,1.81) | 20(8 to 40) | 6.42(2.69 to 12.89) | 105(54 to 181) | 9.84(5.09 to 16.98) | 1.15(0.49,1.82) |
| **Panama** | 1(0 to 1) | 0.12(0.04 to 0.25) | 1(0 to 1) | 0.07(0.03 to 0.13) | -1.85(-3.09,-0.59) | 28(9 to 57) | 5.78(1.95 to 11.70) | 39(18 to 68) | 3.59(1.63 to 6.31) | -1.77(-2.99,-0.53) |
| **Papua New Guinea** | 0(0 to 1) | 0.03(0.01 to 0.11) | 1(0 to 3) | 0.05(0.01 to 0.14) | 1.38(1.01,1.76) | 11(2 to 37) | 1.50(0.27 to 5.26) | 51(11 to 150) | 2.21(0.46 to 6.51) | 1.40(1.02,1.78) |
| **Paraguay** | 0(0 to 1) | 0.04(0.01 to 0.10) | 1(0 to 3) | 0.07(0.02 to 0.16) | 1.82(1.18,2.47) | 14(3 to 35) | 1.86(0.43 to 4.79) | 56(17 to 130) | 3.25(0.96 to 7.49) | 1.80(1.07,2.53) |
| **Peru** | 16(6 to 29) | 0.38(0.15 to 0.70) | 28(13 to 49) | 0.28(0.14 to 0.50) | -0.70(-1.48,0.08) | 766(300 to 1426) | 18.18(7.13 to 33.77) | 1330(636 to 2360) | 13.64(6.53 to 24.20) | -0.72(-1.50,0.07) |
| **Philippines** | 23(10 to 42) | 0.19(0.08 to 0.36) | 59(30 to 100) | 0.22(0.11 to 0.38) | 0.39(0.10,0.69) | 1107(469 to 2068) | 9.29(3.93 to 17.38) | 2869(1477 to 4889) | 10.65(5.48 to 18.14) | 0.40(0.12,0.69) |
| **Poland** | 46(20 to 78) | 0.49(0.21 to 0.82) | 29(18 to 42) | 0.25(0.15 to 0.35) | -2.36(-2.76,-1.96) | 2198(939 to 3684) | 22.93(9.79 to 38.41) | 1356(829 to 1962) | 11.49(7.02 to 16.63) | -2.53(-2.96,-2.11) |
| **Portugal** | 3(1 to 6) | 0.13(0.04 to 0.25) | 2(1 to 4) | 0.06(0.03 to 0.10) | -2.44(-4.10,-0.76) | 160(53 to 308) | 6.09(2.01 to 11.75) | 95(43 to 163) | 2.79(1.27 to 4.82) | -2.50(-4.16,-0.81) |
| **Puerto Rico** | 0(0 to 1) | 0.04(0.00 to 0.13) | 0(0 to 1) | 0.03(0.01 to 0.06) | -0.71(-2.26,0.86) | 19(0 to 62) | 1.96(0.00 to 6.45) | 12(3 to 25) | 1.35(0.32 to 2.77) | -0.76(-2.30,0.80) |
| **Qatar** | 0(0 to 0) | 0.39(0.20 to 0.63) | 1(1 to 2) | 0.19(0.09 to 0.34) | -2.43(-3.73,-1.11) | 12(6 to 20) | 18.67(9.65 to 30.31) | 56(27 to 98) | 9.30(4.47 to 16.30) | -2.38(-3.48,-1.26) |
| **Republic of Korea** | 46(17 to 86) | 0.42(0.16 to 0.79) | 37(20 to 61) | 0.24(0.13 to 0.38) | -1.88(-2.30,-1.46) | 2265(862 to 4261) | 20.51(7.79 to 38.54) | 1775(969 to 2871) | 11.36(6.17 to 18.40) | -1.91(-2.33,-1.49) |
| **Republic of Moldova** | 2(1 to 3) | 0.15(0.05 to 0.31) | 1(0 to 2) | 0.08(0.03 to 0.15) | -2.20(-4.87,0.54) | 78(27 to 164) | 7.02(2.45 to 14.79) | 44(18 to 79) | 3.82(1.56 to 6.89) | -2.16(-4.83,0.58) |
| **Romania** | 16(6 to 29) | 0.28(0.10 to 0.51) | 17(9 to 26) | 0.29(0.16 to 0.45) | 0.23(-0.18,0.64) | 753(263 to 1381) | 13.22(4.62 to 24.26) | 789(437 to 1214) | 13.71(7.56 to 21.12) | 0.18(-0.26,0.62) |
| **Russian Federation** | 100(43 to 162) | 0.28(0.12 to 0.45) | 44(22 to 72) | 0.10(0.05 to 0.16) | -3.37(-4.01,-2.72) | 4825(2063 to 7833) | 13.10(5.60 to 21.26) | 2072(1045 to 3426) | 4.62(2.33 to 7.64) | -3.36(-4.01,-2.71) |
| **Rwanda** | 0(0 to 1) | 0.03(0.01 to 0.06) | 0(0 to 1) | 0.02(0.01 to 0.04) | -1.63(-2.05,-1.20) | 15(6 to 32) | 1.38(0.52 to 2.90) | 23(7 to 53) | 0.82(0.26 to 1.87) | -1.62(-2.05,-1.19) |
| **Saint Kitts and Nevis** | 0(0 to 0) | 0.08(0.02 to 0.17) | 0(0 to 0) | 0.03(0.01 to 0.05) | -3.15(-3.46,-2.84) | 0(0 to 1) | 3.68(0.93 to 8.18) | 0(0 to 0) | 1.25(0.39 to 2.39) | -3.25(-3.56,-2.93) |
| **Saint Lucia** | 0(0 to 0) | 0.19(0.05 to 0.42) | 0(0 to 0) | 0.20(0.07 to 0.36) | 0.21(-0.26,0.68) | 2(1 to 5) | 8.81(2.30 to 19.91) | 5(2 to 9) | 9.37(3.51 to 16.70) | 0.17(-0.29,0.63) |
| **Saint Vincent and the Grenadines** | 0(0 to 0) | 0.12(0.03 to 0.29) | 0(0 to 0) | 0.24(0.09 to 0.46) | 2.08(1.08,3.09) | 1(0 to 2) | 5.73(1.37 to 13.52) | 3(1 to 7) | 11.12(4.02 to 21.41) | 2.05(1.04,3.08) |
| **Samoa** | 0(0 to 0) | 0.01(0.00 to 0.03) | 0(0 to 0) | 0.03(0.01 to 0.08) | 3.01(2.73,3.29) | 0(0 to 0) | 0.51(0.10 to 1.59) | 1(0 to 2) | 1.28(0.28 to 3.78) | 3.01(2.74,3.29) |
| **San Marino** | 0(0 to 0) | 0.18(0.06 to 0.37) | 0(0 to 0) | 0.10(0.03 to 0.21) | -2.20(-2.39,-2.01) | 1(0 to 1) | 8.63(2.67 to 17.28) | 0(0 to 1) | 4.50(1.60 to 9.65) | -2.21(-2.40,-2.01) |
| **Sao Tome and Principe** | 0(0 to 0) | 0.03(0.01 to 0.05) | 0(0 to 0) | 0.06(0.02 to 0.14) | 3.15(1.27,5.07) | 0(0 to 0) | 1.26(0.53 to 2.46) | 1(0 to 3) | 3.05(1.00 to 7.07) | 3.13(1.23,5.07) |
| **Saudi Arabia** | 3(1 to 5) | 0.13(0.06 to 0.22) | 22(11 to 38) | 0.21(0.10 to 0.36) | 1.49(1.28,1.69) | 134(65 to 232) | 6.01(2.91 to 10.41) | 1051(520 to 1831) | 9.72(4.81 to 16.93) | 1.50(1.29,1.70) |
| **Senegal** | 0(0 to 1) | 0.04(0.02 to 0.08) | 1(0 to 2) | 0.03(0.01 to 0.06) | -0.39(-1.14,0.36) | 22(10 to 44) | 1.84(0.79 to 3.62) | 47(18 to 97) | 1.51(0.59 to 3.13) | -0.37(-1.13,0.39) |
| **Serbia** | 16(5 to 30) | 0.65(0.22 to 1.19) | 13(6 to 21) | 0.49(0.23 to 0.79) | -0.98(-1.66,-0.31) | 768(252 to 1403) | 30.74(10.08 to 56.14) | 589(275 to 950) | 22.63(10.54 to 36.58) | -1.04(-1.71,-0.36) |
| **Seychelles** | 0(0 to 0) | 0.07(0.02 to 0.14) | 0(0 to 0) | 0.06(0.02 to 0.14) | -0.07(-0.45,0.31) | 0(0 to 1) | 3.31(0.91 to 6.92) | 1(0 to 2) | 3.18(0.92 to 6.65) | -0.08(-0.44,0.28) |
| **Sierra Leone** | 0(0 to 0) | 0.01(0.01 to 0.03) | 0(0 to 1) | 0.02(0.01 to 0.04) | 0.82(-0.22,1.87) | 5(2 to 10) | 0.69(0.27 to 1.41) | 15(6 to 31) | 0.91(0.36 to 1.81) | 0.87(-0.17,1.93) |
| **Singapore** | 4(1 to 7) | 0.43(0.15 to 0.78) | 2(1 to 4) | 0.12(0.06 to 0.20) | -4.14(-5.49,-2.76) | 180(62 to 330) | 20.46(7.05 to 37.38) | 109(52 to 186) | 5.71(2.70 to 9.70) | -4.15(-5.48,-2.81) |
| **Slovakia** | 4(2 to 8) | 0.32(0.15 to 0.58) | 3(2 to 5) | 0.18(0.09 to 0.30) | -2.04(-2.44,-1.64) | 207(94 to 369) | 15.16(6.92 to 27.03) | 145(75 to 240) | 8.37(4.35 to 13.88) | -2.03(-2.47,-1.58) |
| **Slovenia** | 2(1 to 3) | 0.34(0.16 to 0.57) | 1(0 to 1) | 0.15(0.08 to 0.23) | -2.73(-3.25,-2.21) | 83(38 to 140) | 15.80(7.23 to 26.73) | 42(22 to 65) | 7.17(3.77 to 11.04) | -2.70(-3.21,-2.19) |
| **Solomon Islands** | 0(0 to 0) | 0.03(0.01 to 0.11) | 0(0 to 0) | 0.06(0.02 to 0.16) | 2.23(1.75,2.71) | 1(0 to 3) | 1.56(0.29 to 5.03) | 5(1 to 12) | 3.13(0.90 to 7.83) | 2.26(1.78,2.74) |
| **Somalia** | 0(0 to 0) | 0.01(0.00 to 0.02) | 0(0 to 1) | 0.01(0.00 to 0.02) | -0.54(-1.30,0.22) | 6(3 to 11) | 0.42(0.20 to 0.83) | 13(5 to 28) | 0.36(0.14 to 0.78) | -0.51(-1.26,0.24) |
| **South Africa** | 20(11 to 31) | 0.28(0.15 to 0.42) | 31(17 to 48) | 0.20(0.11 to 0.32) | -1.22(-1.72,-0.72) | 990(536 to 1499) | 13.43(7.29 to 20.28) | 1455(790 to 2295) | 9.52(5.18 to 15.00) | -1.13(-2.00,-0.25) |
| **South Sudan** | 0(0 to 1) | 0.04(0.01 to 0.10) | 0(0 to 1) | 0.02(0.01 to 0.05) | -2.17(-2.93,-1.41) | 16(4 to 40) | 1.88(0.51 to 4.69) | 20(7 to 42) | 1.06(0.40 to 2.21) | -1.95(-2.47,-1.42) |
| **Spain** | 15(6 to 27) | 0.16(0.06 to 0.28) | 14(7 to 23) | 0.09(0.05 to 0.15) | -1.82(-2.24,-1.40) | 724(274 to 1309) | 7.57(2.87 to 13.69) | 646(336 to 1029) | 4.23(2.20 to 6.76) | -1.98(-2.70,-1.26) |
| **Sri Lanka** | 1(0 to 3) | 0.04(0.01 to 0.08) | 6(2 to 11) | 0.09(0.03 to 0.18) | 3.04(2.30,3.79) | 70(24 to 158) | 1.75(0.59 to 3.94) | 270(79 to 528) | 4.35(1.27 to 8.53) | 3.01(2.24,3.77) |
| **Sudan** | 2(1 to 3) | 0.05(0.02 to 0.10) | 14(5 to 28) | 0.15(0.05 to 0.31) | 3.47(3.20,3.75) | 89(35 to 171) | 2.54(1.02 to 4.89) | 675(233 to 1381) | 7.30(2.54 to 14.90) | 3.51(3.23,3.78) |
| **Suriname** | 0(0 to 0) | 0.21(0.05 to 0.44) | 0(0 to 1) | 0.31(0.11 to 0.58) | 1.24(0.07,2.42) | 8(2 to 17) | 9.74(2.59 to 20.84) | 23(8 to 43) | 14.94(5.31 to 27.84) | 1.40(-0.30,3.13) |
| **Sweden** | 4(1 to 9) | 0.17(0.04 to 0.34) | 1(0 to 2) | 0.03(0.01 to 0.06) | -5.78(-6.62,-4.93) | 197(48 to 402) | 7.74(1.90 to 15.83) | 36(9 to 73) | 1.35(0.35 to 2.78) | -5.69(-6.53,-4.84) |
| **Switzerland** | 7(3 to 12) | 0.35(0.14 to 0.61) | 2(1 to 3) | 0.08(0.04 to 0.13) | -5.04(-5.70,-4.38) | 328(133 to 575) | 16.18(6.56 to 28.33) | 96(47 to 156) | 3.84(1.89 to 6.26) | -4.78(-5.62,-3.93) |
| **Syrian Arab Republic** | 5(3 to 10) | 0.28(0.13 to 0.52) | 10(5 to 18) | 0.25(0.11 to 0.46) | -0.46(-1.22,0.30) | 261(124 to 486) | 13.70(6.53 to 25.42) | 471(214 to 872) | 12.03(5.47 to 22.32) | -0.47(-1.21,0.28) |
| **Taiwan (Province of China)** | 20(7 to 39) | 0.43(0.16 to 0.83) | 28(17 to 42) | 0.36(0.21 to 0.53) | -0.57(-1.39,0.25) | 990(359 to 1895) | 20.81(7.52 to 39.90) | 1301(772 to 1943) | 16.88(9.99 to 25.18) | -0.62(-1.43,0.20) |
| **Tajikistan** | 1(0 to 3) | 0.16(0.05 to 0.37) | 3(1 to 5) | 0.11(0.04 to 0.23) | -1.19(-1.91,-0.46) | 64(19 to 147) | 8.02(2.45 to 18.25) | 130(49 to 268) | 5.63(2.14 to 11.56) | -1.17(-1.87,-0.46) |
| **Thailand** | 49(18 to 102) | 0.38(0.14 to 0.79) | 132(68 to 218) | 0.62(0.32 to 1.02) | 1.68(1.32,2.04) | 2329(840 to 4880) | 17.81(6.45 to 37.23) | 6298(3259 to 10402) | 30.23(15.66 to 49.84) | 1.77(1.30,2.24) |
| **Timor-Leste** | 0(0 to 0) | 0.03(0.01 to 0.08) | 0(0 to 1) | 0.08(0.02 to 0.20) | 3.12(2.39,3.85) | 2(0 to 5) | 1.50(0.34 to 3.74) | 10(3 to 25) | 4.00(1.12 to 9.56) | 3.12(2.38,3.87) |
| **Togo** | 0(0 to 0) | 0.03(0.01 to 0.05) | 1(0 to 1) | 0.03(0.01 to 0.06) | -0.09(-1.01,0.84) | 9(3 to 16) | 1.38(0.57 to 2.57) | 26(10 to 54) | 1.35(0.52 to 2.83) | -0.06(-0.98,0.87) |
| **Tokelau** | 0(0 to 0) | 0.06(0.00 to 0.22) | 0(0 to 0) | 0.06(0.00 to 0.18) | 0.42(-0.06,0.91) | 0(0 to 0) | 2.70(0.09 to 10.38) | 0(0 to 0) | 3.11(0.03 to 8.91) | 0.49(0.04,0.95) |
| **Tonga** | 0(0 to 0) | 0.04(0.01 to 0.11) | 0(0 to 0) | 0.08(0.02 to 0.21) | 2.41(2.24,2.58) | 0(0 to 1) | 1.87(0.39 to 5.37) | 1(0 to 2) | 3.92(0.98 to 10.12) | 2.42(2.26,2.57) |
| **Trinidad and Tobago** | 0(0 to 1) | 0.14(0.02 to 0.32) | 1(0 to 1) | 0.16(0.05 to 0.32) | 0.29(-0.18,0.76) | 17(3 to 38) | 6.75(1.17 to 14.84) | 30(10 to 59) | 7.53(2.60 to 14.98) | 0.32(-0.14,0.79) |
| **Tunisia** | 1(0 to 1) | 0.06(0.03 to 0.10) | 4(2 to 6) | 0.10(0.04 to 0.18) | 1.89(1.68,2.09) | 40(19 to 71) | 2.62(1.22 to 4.64) | 167(74 to 299) | 4.66(2.08 to 8.38) | 1.88(1.68,2.09) |
| **Turkey** | 38(16 to 69) | 0.34(0.14 to 0.61) | 76(39 to 125) | 0.31(0.16 to 0.51) | -0.35(-0.75,0.05) | 1870(772 to 3360) | 16.44(6.81 to 29.47) | 3603(1873 to 5940) | 14.77(7.68 to 24.36) | -0.35(-0.76,0.07) |
| **Turkmenistan** | 1(0 to 2) | 0.20(0.05 to 0.38) | 2(1 to 3) | 0.15(0.07 to 0.27) | -0.87(-2.06,0.33) | 62(17 to 120) | 9.77(2.67 to 18.76) | 93(45 to 160) | 7.51(3.60 to 12.97) | -0.89(-2.05,0.29) |
| **Tuvalu** | 0(0 to 0) | 0.01(0.00 to 0.03) | 0(0 to 0) | 0.03(0.01 to 0.07) | 2.69(2.51,2.87) | 0(0 to 0) | 0.58(0.15 to 1.54) | 0(0 to 0) | 1.31(0.41 to 3.32) | 2.70(2.52,2.88) |
| **Uganda** | 0(0 to 1) | 0.01(0.01 to 0.03) | 2(1 to 5) | 0.03(0.01 to 0.07) | 3.01(2.59,3.42) | 16(7 to 31) | 0.67(0.30 to 1.26) | 118(46 to 232) | 1.65(0.66 to 3.25) | 3.03(2.62,3.45) |
| **Ukraine** | 62(27 to 102) | 0.46(0.20 to 0.75) | 20(8 to 40) | 0.14(0.06 to 0.30) | -3.74(-4.32,-3.15) | 2961(1304 to 4896) | 21.76(9.59 to 36.03) | 932(384 to 1927) | 6.91(2.84 to 14.31) | -3.71(-4.30,-3.12) |
| **United Arab Emirates** | 1(0 to 2) | 0.34(0.14 to 0.69) | 5(2 to 9) | 0.23(0.11 to 0.41) | -1.28(-1.75,-0.81) | 38(16 to 79) | 16.03(6.75 to 32.97) | 251(118 to 452) | 11.41(5.31 to 20.54) | -1.12(-1.76,-0.47) |
| **United Kingdom** | 57(23 to 99) | 0.37(0.15 to 0.64) | 19(11 to 29) | 0.10(0.06 to 0.16) | -4.12(-4.43,-3.81) | 2635(1076 to 4566) | 17.03(6.95 to 29.51) | 893(491 to 1368) | 4.87(2.68 to 7.46) | -4.07(-4.39,-3.74) |
| **United Republic of Tanzania** | 0(0 to 1) | 0.01(0.01 to 0.02) | 2(1 to 5) | 0.02(0.01 to 0.04) | 2.17(1.57,2.78) | 22(10 to 42) | 0.55(0.26 to 1.03) | 115(44 to 241) | 1.01(0.39 to 2.10) | 2.18(1.58,2.79) |
| **United States of America** | 298(118 to 539) | 0.44(0.17 to 0.79) | 48(21 to 84) | 0.06(0.02 to 0.10) | -6.22(-6.64,-5.80) | 13874(5488 to 25105) | 20.32(8.04 to 36.79) | 2237(955 to 3877) | 2.59(1.11 to 4.49) | -6.21(-6.60,-5.82) |
| **United States Virgin Islands** | 0(0 to 0) | 0.08(0.00 to 0.24) | 0(0 to 0) | 0.06(0.02 to 0.13) | -0.74(-1.58,0.11) | 1(0 to 4) | 3.90(0.09 to 11.45) | 1(0 to 1) | 3.03(1.00 to 6.26) | -0.63(-1.42,0.17) |
| **Uruguay** | 1(0 to 2) | 0.13(0.04 to 0.25) | 1(1 to 3) | 0.15(0.05 to 0.29) | 0.44(-0.35,1.24) | 47(16 to 91) | 6.18(2.07 to 11.96) | 67(24 to 130) | 7.05(2.56 to 13.72) | 0.46(-0.33,1.25) |
| **Uzbekistan** | 6(2 to 13) | 0.20(0.07 to 0.41) | 17(9 to 27) | 0.18(0.10 to 0.29) | -0.23(-0.96,0.50) | 326(110 to 676) | 9.52(3.20 to 19.83) | 826(432 to 1332) | 8.92(4.67 to 14.38) | -0.18(-0.87,0.52) |
| **Vanuatu** | 0(0 to 0) | 0.01(0.00 to 0.05) | 0(0 to 0) | 0.03(0.01 to 0.07) | 2.21(1.79,2.64) | 0(0 to 1) | 0.66(0.12 to 2.24) | 1(0 to 2) | 1.31(0.36 to 3.43) | 2.21(1.93,2.49) |
| **Venezuela (Bolivarian Republic of)** | 14(6 to 24) | 0.39(0.17 to 0.66) | 14(6 to 27) | 0.18(0.08 to 0.35) | -2.37(-3.19,-1.55) | 687(297 to 1165) | 18.63(8.09 to 31.58) | 680(303 to 1294) | 8.69(3.87 to 16.55) | -2.36(-3.21,-1.51) |
| **Viet Nam** | 3(1 to 7) | 0.03(0.01 to 0.07) | 49(18 to 100) | 0.17(0.06 to 0.35) | 5.72(5.46,5.98) | 163(60 to 355) | 1.43(0.53 to 3.11) | 2267(846 to 4677) | 7.85(2.93 to 16.19) | 5.71(5.49,5.92) |
| **Yemen** | 1(0 to 2) | 0.05(0.02 to 0.11) | 7(3 to 16) | 0.11(0.04 to 0.24) | 2.42(1.90,2.94) | 45(16 to 97) | 2.37(0.85 to 5.04) | 353(130 to 760) | 5.34(1.99 to 11.50) | 2.45(1.92,2.97) |
| **Zambia** | 0(0 to 1) | 0.03(0.01 to 0.06) | 2(1 to 5) | 0.06(0.02 to 0.14) | 2.23(1.96,2.50) | 17(7 to 32) | 1.41(0.63 to 2.72) | 96(30 to 241) | 2.74(0.86 to 6.82) | 2.24(1.98,2.50) |
| **Zimbabwe** | 1(0 to 1) | 0.03(0.01 to 0.07) | 2(1 to 4) | 0.05(0.02 to 0.12) | 1.46(0.37,2.56) | 25(10 to 50) | 1.63(0.67 to 3.29) | 82(34 to 176) | 2.54(1.04 to 5.47) | 1.48(0.37,2.59) |
| **Marshall Islands** | 0(0 to 0) | 0.04(0.01 to 0.13) | 0(0 to 0) | 0.08(0.02 to 0.23) | 1.88(1.60,2.16) | 0(0 to 0) | 2.08(0.42 to 6.18) | 1(0 to 2) | 3.71(0.79 to 10.91) | 1.90(1.64,2.15) |
| **Mauritania** | 0(0 to 0) | 0.05(0.02 to 0.10) | 1(0 to 1) | 0.08(0.03 to 0.17) | 1.46(0.90,2.02) | 8(3 to 16) | 2.39(0.98 to 4.81) | 32(13 to 66) | 3.88(1.57 to 8.00) | 1.50(0.93,2.06) |
| **Mauritius** | 0(0 to 0) | 0.06(0.02 to 0.11) | 0(0 to 0) | 0.06(0.02 to 0.11) | -0.04(-1.91,1.87) | 7(3 to 13) | 2.68(1.05 to 5.12) | 10(3 to 19) | 2.82(0.89 to 5.28) | -0.03(-1.84,1.82) |
| **Mexico** | 36(17 to 56) | 0.22(0.11 to 0.35) | 30(17 to 46) | 0.08(0.04 to 0.12) | -3.23(-4.18,-2.27) | 1751(854 to 2734) | 10.78(5.25 to 16.82) | 1420(788 to 2199) | 3.84(2.13 to 5.95) | -3.25(-4.20,-2.29) |
| **Micronesia (Federated States of)** | 0(0 to 0) | 0.05(0.01 to 0.15) | 0(0 to 0) | 0.09(0.02 to 0.26) | 1.94(1.80,2.08) | 0(0 to 1) | 2.38(0.50 to 7.21) | 1(0 to 3) | 4.32(1.03 to 12.16) | 1.95(1.82,2.07) |
| **Monaco** | 0(0 to 0) | 0.25(0.03 to 0.57) | 0(0 to 0) | 0.44(0.18 to 0.82) | 1.87(1.26,2.48) | 1(0 to 3) | 12.02(1.65 to 26.96) | 2(1 to 4) | 21.08(8.50 to 39.14) | 1.89(1.28,2.51) |
| **Mongolia** | 0(0 to 1) | 0.11(0.04 to 0.25) | 3(1 to 5) | 0.29(0.11 to 0.49) | 2.94(2.00,3.89) | 19(7 to 42) | 5.49(1.92 to 11.93) | 129(50 to 218) | 13.96(5.37 to 23.59) | 2.94(2.02,3.88) |
| **Montenegro** | 1(0 to 2) | 0.68(0.25 to 1.20) | 1(0 to 1) | 0.46(0.21 to 0.74) | -1.15(-2.15,-0.14) | 47(17 to 84) | 31.71(11.60 to 56.48) | 39(18 to 62) | 21.61(9.96 to 34.56) | -1.14(-2.07,-0.21) |
| **Morocco** | 1(0 to 2) | 0.02(0.01 to 0.04) | 5(2 to 9) | 0.05(0.02 to 0.09) | 3.20(2.92,3.47) | 42(19 to 79) | 0.88(0.40 to 1.68) | 231(107 to 444) | 2.30(1.07 to 4.42) | 3.18(2.91,3.45) |
| **Mozambique** | 0(0 to 0) | 0.01(0.00 to 0.01) | 0(0 to 1) | 0.01(0.00 to 0.02) | 1.47(1.04,1.90) | 6(3 to 10) | 0.24(0.12 to 0.42) | 19(8 to 39) | 0.37(0.15 to 0.74) | 1.47(1.09,1.86) |
| **Myanmar** | 8(3 to 19) | 0.11(0.04 to 0.25) | 29(11 to 59) | 0.19(0.07 to 0.39) | 1.93(1.73,2.12) | 414(137 to 950) | 5.23(1.74 to 11.93) | 1397(504 to 2857) | 9.20(3.31 to 18.82) | 1.93(1.73,2.12) |
| **Namibia** | 0(0 to 0) | 0.03(0.01 to 0.07) | 0(0 to 1) | 0.07(0.02 to 0.15) | 2.40(1.81,3.01) | 4(1 to 8) | 1.56(0.51 to 3.39) | 18(6 to 39) | 3.18(1.02 to 6.96) | 2.41(1.81,3.01) |
| **Nauru** | 0(0 to 0) | 0.09(0.00 to 0.41) | 0(0 to 0) | 0.11(0.01 to 0.31) | 0.44(0.12,0.76) | 0(0 to 0) | 4.26(0.00 to 19.27) | 0(0 to 0) | 4.98(0.60 to 14.51) | 0.45(0.13,0.78) |
| **Nepal** | 1(0 to 2) | 0.03(0.01 to 0.06) | 5(2 to 10) | 0.06(0.03 to 0.12) | 2.15(1.32,2.98) | 50(17 to 110) | 1.40(0.49 to 3.09) | 242(104 to 469) | 3.06(1.32 to 5.90) | 2.15(1.32,3.00) |
| **Netherlands** | 21(9 to 36) | 0.52(0.23 to 0.88) | 8(5 to 13) | 0.18(0.10 to 0.28) | -3.43(-4.10,-2.76) | 1001(442 to 1701) | 24.12(10.65 to 41.00) | 387(214 to 597) | 8.67(4.78 to 13.40) | -3.42(-4.08,-2.76) |
| **New Zealand** | 1(0 to 3) | 0.12(0.00 to 0.36) | 1(0 to 2) | 0.09(0.03 to 0.16) | -1.06(-1.43,-0.69) | 47(1 to 145) | 5.39(0.14 to 16.52) | 54(19 to 100) | 4.01(1.39 to 7.43) | -1.04(-1.41,-0.67) |
| **Nicaragua** | 0(0 to 0) | 0.04(0.01 to 0.08) | 1(0 to 1) | 0.03(0.01 to 0.07) | -0.51(-1.20,0.18) | 12(4 to 25) | 1.86(0.69 to 3.95) | 27(9 to 57) | 1.56(0.56 to 3.35) | -0.54(-1.23,0.16) |
| **Niger** | 0(0 to 1) | 0.02(0.01 to 0.04) | 1(0 to 1) | 0.01(0.00 to 0.04) | -0.60(-1.89,0.70) | 11(4 to 25) | 0.88(0.31 to 1.99) | 25(8 to 61) | 0.70(0.24 to 1.70) | -0.58(-1.85,0.72) |
| **Nigeria** | 1(0 to 2) | 0.01(0.00 to 0.02) | 6(2 to 12) | 0.01(0.01 to 0.03) | 1.90(1.13,2.67) | 48(18 to 97) | 0.36(0.14 to 0.72) | 279(101 to 569) | 0.66(0.24 to 1.34) | 1.92(1.14,2.70) |
| **Niue** | 0(0 to 0) | 0.06(0.02 to 0.16) | 0(0 to 0) | 0.07(0.01 to 0.17) | 0.48(0.17,0.79) | 0(0 to 0) | 2.93(0.73 to 7.39) | 0(0 to 0) | 3.51(0.48 to 8.48) | 0.59(0.27,0.92) |
| **North Macedonia** | 2(1 to 3) | 0.36(0.13 to 0.63) | 2(1 to 4) | 0.34(0.18 to 0.55) | -0.23(-0.88,0.42) | 86(32 to 150) | 16.96(6.34 to 29.69) | 108(56 to 173) | 16.05(8.34 to 25.81) | -0.26(-0.90,0.38) |
| **Northern Mariana Islands** | 0(0 to 0) | 0.15(0.00 to 0.42) | 0(0 to 0) | 0.14(0.06 to 0.25) | -0.51(-1.27,0.26) | 1(0 to 2) | 6.98(0.00 to 19.94) | 1(0 to 2) | 6.47(2.66 to 12.09) | -0.42(-0.99,0.16) |
| **Norway** | 2(0 to 3) | 0.16(0.04 to 0.30) | 1(0 to 1) | 0.05(0.02 to 0.09) | -4.00(-4.81,-3.18) | 83(23 to 159) | 7.30(2.02 to 14.02) | 31(11 to 60) | 2.13(0.76 to 4.08) | -4.00(-4.81,-3.18) |
| **Oman** | 0(0 to 0) | 0.06(0.03 to 0.13) | 1(0 to 1) | 0.05(0.03 to 0.10) | -0.53(-1.10,0.04) | 7(3 to 14) | 3.01(1.26 to 5.94) | 26(12 to 48) | 2.60(1.26 to 4.87) | -0.48(-1.03,0.08) |
| **Pakistan** | 8(3 to 15) | 0.04(0.02 to 0.08) | 55(23 to 107) | 0.11(0.05 to 0.21) | 2.92(1.99,3.85) | 362(156 to 712) | 1.95(0.84 to 3.82) | 2683(1132 to 5199) | 5.16(2.18 to 10.00) | 2.94(2.02,3.88) |
| **Palau** | 0(0 to 0) | 0.18(0.00 to 0.55) | 0(0 to 0) | 0.17(0.01 to 0.39) | -0.33(-1.29,0.63) | 0(0 to 1) | 8.53(0.04 to 26.08) | 0(0 to 1) | 8.13(0.71 to 18.95) | -0.30(-1.25,0.67) |
| **Palestine** | 0(0 to 1) | 0.13(0.06 to 0.27) | 2(1 to 4) | 0.20(0.11 to 0.35) | 1.13(0.44,1.81) | 20(8 to 40) | 6.42(2.69 to 12.89) | 105(54 to 181) | 9.84(5.09 to 16.98) | 1.15(0.49,1.82) |
| **Panama** | 1(0 to 1) | 0.12(0.04 to 0.25) | 1(0 to 1) | 0.07(0.03 to 0.13) | -1.85(-3.09,-0.59) | 28(9 to 57) | 5.78(1.95 to 11.70) | 39(18 to 68) | 3.59(1.63 to 6.31) | -1.77(-2.99,-0.53) |
| **Papua New Guinea** | 0(0 to 1) | 0.03(0.01 to 0.11) | 1(0 to 3) | 0.05(0.01 to 0.14) | 1.38(1.01,1.76) | 11(2 to 37) | 1.50(0.27 to 5.26) | 51(11 to 150) | 2.21(0.46 to 6.51) | 1.40(1.02,1.78) |
| **Paraguay** | 0(0 to 1) | 0.04(0.01 to 0.10) | 1(0 to 3) | 0.07(0.02 to 0.16) | 1.82(1.18,2.47) | 14(3 to 35) | 1.86(0.43 to 4.79) | 56(17 to 130) | 3.25(0.96 to 7.49) | 1.80(1.07,2.53) |
| **Peru** | 16(6 to 29) | 0.38(0.15 to 0.70) | 28(13 to 49) | 0.28(0.14 to 0.50) | -0.70(-1.48,0.08) | 766(300 to 1426) | 18.18(7.13 to 33.77) | 1330(636 to 2360) | 13.64(6.53 to 24.20) | -0.72(-1.50,0.07) |
| **Philippines** | 23(10 to 42) | 0.19(0.08 to 0.36) | 59(30 to 100) | 0.22(0.11 to 0.38) | 0.39(0.10,0.69) | 1107(469 to 2068) | 9.29(3.93 to 17.38) | 2869(1477 to 4889) | 10.65(5.48 to 18.14) | 0.40(0.12,0.69) |
| **Poland** | 46(20 to 78) | 0.49(0.21 to 0.82) | 29(18 to 42) | 0.25(0.15 to 0.35) | -2.36(-2.76,-1.96) | 2198(939 to 3684) | 22.93(9.79 to 38.41) | 1356(829 to 1962) | 11.49(7.02 to 16.63) | -2.53(-2.96,-2.11) |
| **Portugal** | 3(1 to 6) | 0.13(0.04 to 0.25) | 2(1 to 4) | 0.06(0.03 to 0.10) | -2.44(-4.10,-0.76) | 160(53 to 308) | 6.09(2.01 to 11.75) | 95(43 to 163) | 2.79(1.27 to 4.82) | -2.50(-4.16,-0.81) |
| **Puerto Rico** | 0(0 to 1) | 0.04(0.00 to 0.13) | 0(0 to 1) | 0.03(0.01 to 0.06) | -0.71(-2.26,0.86) | 19(0 to 62) | 1.96(0.00 to 6.45) | 12(3 to 25) | 1.35(0.32 to 2.77) | -0.76(-2.30,0.80) |
| **Qatar** | 0(0 to 0) | 0.39(0.20 to 0.63) | 1(1 to 2) | 0.19(0.09 to 0.34) | -2.43(-3.73,-1.11) | 12(6 to 20) | 18.67(9.65 to 30.31) | 56(27 to 98) | 9.30(4.47 to 16.30) | -2.38(-3.48,-1.26) |
| **Republic of Korea** | 46(17 to 86) | 0.42(0.16 to 0.79) | 37(20 to 61) | 0.24(0.13 to 0.38) | -1.88(-2.30,-1.46) | 2265(862 to 4261) | 20.51(7.79 to 38.54) | 1775(969 to 2871) | 11.36(6.17 to 18.40) | -1.91(-2.33,-1.49) |
| **Republic of Moldova** | 2(1 to 3) | 0.15(0.05 to 0.31) | 1(0 to 2) | 0.08(0.03 to 0.15) | -2.20(-4.87,0.54) | 78(27 to 164) | 7.02(2.45 to 14.79) | 44(18 to 79) | 3.82(1.56 to 6.89) | -2.16(-4.83,0.58) |
| **Romania** | 16(6 to 29) | 0.28(0.10 to 0.51) | 17(9 to 26) | 0.29(0.16 to 0.45) | 0.23(-0.18,0.64) | 753(263 to 1381) | 13.22(4.62 to 24.26) | 789(437 to 1214) | 13.71(7.56 to 21.12) | 0.18(-0.26,0.62) |
| **Russian Federation** | 100(43 to 162) | 0.28(0.12 to 0.45) | 44(22 to 72) | 0.10(0.05 to 0.16) | -3.37(-4.01,-2.72) | 4825(2063 to 7833) | 13.10(5.60 to 21.26) | 2072(1045 to 3426) | 4.62(2.33 to 7.64) | -3.36(-4.01,-2.71) |
| **Rwanda** | 0(0 to 1) | 0.03(0.01 to 0.06) | 0(0 to 1) | 0.02(0.01 to 0.04) | -1.63(-2.05,-1.20) | 15(6 to 32) | 1.38(0.52 to 2.90) | 23(7 to 53) | 0.82(0.26 to 1.87) | -1.62(-2.05,-1.19) |
| **Saint Kitts and Nevis** | 0(0 to 0) | 0.08(0.02 to 0.17) | 0(0 to 0) | 0.03(0.01 to 0.05) | -3.15(-3.46,-2.84) | 0(0 to 1) | 3.68(0.93 to 8.18) | 0(0 to 0) | 1.25(0.39 to 2.39) | -3.25(-3.56,-2.93) |
| **Saint Lucia** | 0(0 to 0) | 0.19(0.05 to 0.42) | 0(0 to 0) | 0.20(0.07 to 0.36) | 0.21(-0.26,0.68) | 2(1 to 5) | 8.81(2.30 to 19.91) | 5(2 to 9) | 9.37(3.51 to 16.70) | 0.17(-0.29,0.63) |
| **Saint Vincent and the Grenadines** | 0(0 to 0) | 0.12(0.03 to 0.29) | 0(0 to 0) | 0.24(0.09 to 0.46) | 2.08(1.08,3.09) | 1(0 to 2) | 5.73(1.37 to 13.52) | 3(1 to 7) | 11.12(4.02 to 21.41) | 2.05(1.04,3.08) |
| **Samoa** | 0(0 to 0) | 0.01(0.00 to 0.03) | 0(0 to 0) | 0.03(0.01 to 0.08) | 3.01(2.73,3.29) | 0(0 to 0) | 0.51(0.10 to 1.59) | 1(0 to 2) | 1.28(0.28 to 3.78) | 3.01(2.74,3.29) |
| **San Marino** | 0(0 to 0) | 0.18(0.06 to 0.37) | 0(0 to 0) | 0.10(0.03 to 0.21) | -2.20(-2.39,-2.01) | 1(0 to 1) | 8.63(2.67 to 17.28) | 0(0 to 1) | 4.50(1.60 to 9.65) | -2.21(-2.40,-2.01) |
| **Sao Tome and Principe** | 0(0 to 0) | 0.03(0.01 to 0.05) | 0(0 to 0) | 0.06(0.02 to 0.14) | 3.15(1.27,5.07) | 0(0 to 0) | 1.26(0.53 to 2.46) | 1(0 to 3) | 3.05(1.00 to 7.07) | 3.13(1.23,5.07) |
| **Saudi Arabia** | 3(1 to 5) | 0.13(0.06 to 0.22) | 22(11 to 38) | 0.21(0.10 to 0.36) | 1.49(1.28,1.69) | 134(65 to 232) | 6.01(2.91 to 10.41) | 1051(520 to 1831) | 9.72(4.81 to 16.93) | 1.50(1.29,1.70) |
| **Senegal** | 0(0 to 1) | 0.04(0.02 to 0.08) | 1(0 to 2) | 0.03(0.01 to 0.06) | -0.39(-1.14,0.36) | 22(10 to 44) | 1.84(0.79 to 3.62) | 47(18 to 97) | 1.51(0.59 to 3.13) | -0.37(-1.13,0.39) |
| **Serbia** | 16(5 to 30) | 0.65(0.22 to 1.19) | 13(6 to 21) | 0.49(0.23 to 0.79) | -0.98(-1.66,-0.31) | 768(252 to 1403) | 30.74(10.08 to 56.14) | 589(275 to 950) | 22.63(10.54 to 36.58) | -1.04(-1.71,-0.36) |
| **Seychelles** | 0(0 to 0) | 0.07(0.02 to 0.14) | 0(0 to 0) | 0.06(0.02 to 0.14) | -0.07(-0.45,0.31) | 0(0 to 1) | 3.31(0.91 to 6.92) | 1(0 to 2) | 3.18(0.92 to 6.65) | -0.08(-0.44,0.28) |
| **Sierra Leone** | 0(0 to 0) | 0.01(0.01 to 0.03) | 0(0 to 1) | 0.02(0.01 to 0.04) | 0.82(-0.22,1.87) | 5(2 to 10) | 0.69(0.27 to 1.41) | 15(6 to 31) | 0.91(0.36 to 1.81) | 0.87(-0.17,1.93) |
| **Singapore** | 4(1 to 7) | 0.43(0.15 to 0.78) | 2(1 to 4) | 0.12(0.06 to 0.20) | -4.14(-5.49,-2.76) | 180(62 to 330) | 20.46(7.05 to 37.38) | 109(52 to 186) | 5.71(2.70 to 9.70) | -4.15(-5.48,-2.81) |
| **Slovakia** | 4(2 to 8) | 0.32(0.15 to 0.58) | 3(2 to 5) | 0.18(0.09 to 0.30) | -2.04(-2.44,-1.64) | 207(94 to 369) | 15.16(6.92 to 27.03) | 145(75 to 240) | 8.37(4.35 to 13.88) | -2.03(-2.47,-1.58) |
| **Slovenia** | 2(1 to 3) | 0.34(0.16 to 0.57) | 1(0 to 1) | 0.15(0.08 to 0.23) | -2.73(-3.25,-2.21) | 83(38 to 140) | 15.80(7.23 to 26.73) | 42(22 to 65) | 7.17(3.77 to 11.04) | -2.70(-3.21,-2.19) |
| **Solomon Islands** | 0(0 to 0) | 0.03(0.01 to 0.11) | 0(0 to 0) | 0.06(0.02 to 0.16) | 2.23(1.75,2.71) | 1(0 to 3) | 1.56(0.29 to 5.03) | 5(1 to 12) | 3.13(0.90 to 7.83) | 2.26(1.78,2.74) |
| **Somalia** | 0(0 to 0) | 0.01(0.00 to 0.02) | 0(0 to 1) | 0.01(0.00 to 0.02) | -0.54(-1.30,0.22) | 6(3 to 11) | 0.42(0.20 to 0.83) | 13(5 to 28) | 0.36(0.14 to 0.78) | -0.51(-1.26,0.24) |
| **South Africa** | 20(11 to 31) | 0.28(0.15 to 0.42) | 31(17 to 48) | 0.20(0.11 to 0.32) | -1.22(-1.72,-0.72) | 990(536 to 1499) | 13.43(7.29 to 20.28) | 1455(790 to 2295) | 9.52(5.18 to 15.00) | -1.13(-2.00,-0.25) |
| **South Sudan** | 0(0 to 1) | 0.04(0.01 to 0.10) | 0(0 to 1) | 0.02(0.01 to 0.05) | -2.17(-2.93,-1.41) | 16(4 to 40) | 1.88(0.51 to 4.69) | 20(7 to 42) | 1.06(0.40 to 2.21) | -1.95(-2.47,-1.42) |
| **Spain** | 15(6 to 27) | 0.16(0.06 to 0.28) | 14(7 to 23) | 0.09(0.05 to 0.15) | -1.82(-2.24,-1.40) | 724(274 to 1309) | 7.57(2.87 to 13.69) | 646(336 to 1029) | 4.23(2.20 to 6.76) | -1.98(-2.70,-1.26) |
| **Sri Lanka** | 1(0 to 3) | 0.04(0.01 to 0.08) | 6(2 to 11) | 0.09(0.03 to 0.18) | 3.04(2.30,3.79) | 70(24 to 158) | 1.75(0.59 to 3.94) | 270(79 to 528) | 4.35(1.27 to 8.53) | 3.01(2.24,3.77) |
| **Sudan** | 2(1 to 3) | 0.05(0.02 to 0.10) | 14(5 to 28) | 0.15(0.05 to 0.31) | 3.47(3.20,3.75) | 89(35 to 171) | 2.54(1.02 to 4.89) | 675(233 to 1381) | 7.30(2.54 to 14.90) | 3.51(3.23,3.78) |
| **Suriname** | 0(0 to 0) | 0.21(0.05 to 0.44) | 0(0 to 1) | 0.31(0.11 to 0.58) | 1.24(0.07,2.42) | 8(2 to 17) | 9.74(2.59 to 20.84) | 23(8 to 43) | 14.94(5.31 to 27.84) | 1.40(-0.30,3.13) |
| **Sweden** | 4(1 to 9) | 0.17(0.04 to 0.34) | 1(0 to 2) | 0.03(0.01 to 0.06) | -5.78(-6.62,-4.93) | 197(48 to 402) | 7.74(1.90 to 15.83) | 36(9 to 73) | 1.35(0.35 to 2.78) | -5.69(-6.53,-4.84) |
| **Switzerland** | 7(3 to 12) | 0.35(0.14 to 0.61) | 2(1 to 3) | 0.08(0.04 to 0.13) | -5.04(-5.70,-4.38) | 328(133 to 575) | 16.18(6.56 to 28.33) | 96(47 to 156) | 3.84(1.89 to 6.26) | -4.78(-5.62,-3.93) |
| **Syrian Arab Republic** | 5(3 to 10) | 0.28(0.13 to 0.52) | 10(5 to 18) | 0.25(0.11 to 0.46) | -0.46(-1.22,0.30) | 261(124 to 486) | 13.70(6.53 to 25.42) | 471(214 to 872) | 12.03(5.47 to 22.32) | -0.47(-1.21,0.28) |
| **Taiwan (Province of China)** | 20(7 to 39) | 0.43(0.16 to 0.83) | 28(17 to 42) | 0.36(0.21 to 0.53) | -0.57(-1.39,0.25) | 990(359 to 1895) | 20.81(7.52 to 39.90) | 1301(772 to 1943) | 16.88(9.99 to 25.18) | -0.62(-1.43,0.20) |
| **Tajikistan** | 1(0 to 3) | 0.16(0.05 to 0.37) | 3(1 to 5) | 0.11(0.04 to 0.23) | -1.19(-1.91,-0.46) | 64(19 to 147) | 8.02(2.45 to 18.25) | 130(49 to 268) | 5.63(2.14 to 11.56) | -1.17(-1.87,-0.46) |
| **Thailand** | 49(18 to 102) | 0.38(0.14 to 0.79) | 132(68 to 218) | 0.62(0.32 to 1.02) | 1.68(1.32,2.04) | 2329(840 to 4880) | 17.81(6.45 to 37.23) | 6298(3259 to 10402) | 30.23(15.66 to 49.84) | 1.77(1.30,2.24) |
| **Timor-Leste** | 0(0 to 0) | 0.03(0.01 to 0.08) | 0(0 to 1) | 0.08(0.02 to 0.20) | 3.12(2.39,3.85) | 2(0 to 5) | 1.50(0.34 to 3.74) | 10(3 to 25) | 4.00(1.12 to 9.56) | 3.12(2.38,3.87) |
| **Togo** | 0(0 to 0) | 0.03(0.01 to 0.05) | 1(0 to 1) | 0.03(0.01 to 0.06) | -0.09(-1.01,0.84) | 9(3 to 16) | 1.38(0.57 to 2.57) | 26(10 to 54) | 1.35(0.52 to 2.83) | -0.06(-0.98,0.87) |
| **Tokelau** | 0(0 to 0) | 0.06(0.00 to 0.22) | 0(0 to 0) | 0.06(0.00 to 0.18) | 0.42(-0.06,0.91) | 0(0 to 0) | 2.70(0.09 to 10.38) | 0(0 to 0) | 3.11(0.03 to 8.91) | 0.49(0.04,0.95) |
| **Tonga** | 0(0 to 0) | 0.04(0.01 to 0.11) | 0(0 to 0) | 0.08(0.02 to 0.21) | 2.41(2.24,2.58) | 0(0 to 1) | 1.87(0.39 to 5.37) | 1(0 to 2) | 3.92(0.98 to 10.12) | 2.42(2.26,2.57) |
| **Trinidad and Tobago** | 0(0 to 1) | 0.14(0.02 to 0.32) | 1(0 to 1) | 0.16(0.05 to 0.32) | 0.29(-0.18,0.76) | 17(3 to 38) | 6.75(1.17 to 14.84) | 30(10 to 59) | 7.53(2.60 to 14.98) | 0.32(-0.14,0.79) |
| **Tunisia** | 1(0 to 1) | 0.06(0.03 to 0.10) | 4(2 to 6) | 0.10(0.04 to 0.18) | 1.89(1.68,2.09) | 40(19 to 71) | 2.62(1.22 to 4.64) | 167(74 to 299) | 4.66(2.08 to 8.38) | 1.88(1.68,2.09) |
| **Turkey** | 38(16 to 69) | 0.34(0.14 to 0.61) | 76(39 to 125) | 0.31(0.16 to 0.51) | -0.35(-0.75,0.05) | 1870(772 to 3360) | 16.44(6.81 to 29.47) | 3603(1873 to 5940) | 14.77(7.68 to 24.36) | -0.35(-0.76,0.07) |
| **Turkmenistan** | 1(0 to 2) | 0.20(0.05 to 0.38) | 2(1 to 3) | 0.15(0.07 to 0.27) | -0.87(-2.06,0.33) | 62(17 to 120) | 9.77(2.67 to 18.76) | 93(45 to 160) | 7.51(3.60 to 12.97) | -0.89(-2.05,0.29) |
| **Tuvalu** | 0(0 to 0) | 0.01(0.00 to 0.03) | 0(0 to 0) | 0.03(0.01 to 0.07) | 2.69(2.51,2.87) | 0(0 to 0) | 0.58(0.15 to 1.54) | 0(0 to 0) | 1.31(0.41 to 3.32) | 2.70(2.52,2.88) |
| **Uganda** | 0(0 to 1) | 0.01(0.01 to 0.03) | 2(1 to 5) | 0.03(0.01 to 0.07) | 3.01(2.59,3.42) | 16(7 to 31) | 0.67(0.30 to 1.26) | 118(46 to 232) | 1.65(0.66 to 3.25) | 3.03(2.62,3.45) |
| **Ukraine** | 62(27 to 102) | 0.46(0.20 to 0.75) | 20(8 to 40) | 0.14(0.06 to 0.30) | -3.74(-4.32,-3.15) | 2961(1304 to 4896) | 21.76(9.59 to 36.03) | 932(384 to 1927) | 6.91(2.84 to 14.31) | -3.71(-4.30,-3.12) |
| **United Arab Emirates** | 1(0 to 2) | 0.34(0.14 to 0.69) | 5(2 to 9) | 0.23(0.11 to 0.41) | -1.28(-1.75,-0.81) | 38(16 to 79) | 16.03(6.75 to 32.97) | 251(118 to 452) | 11.41(5.31 to 20.54) | -1.12(-1.76,-0.47) |
| **United Kingdom** | 57(23 to 99) | 0.37(0.15 to 0.64) | 19(11 to 29) | 0.10(0.06 to 0.16) | -4.12(-4.43,-3.81) | 2635(1076 to 4566) | 17.03(6.95 to 29.51) | 893(491 to 1368) | 4.87(2.68 to 7.46) | -4.07(-4.39,-3.74) |
| **United Republic of Tanzania** | 0(0 to 1) | 0.01(0.01 to 0.02) | 2(1 to 5) | 0.02(0.01 to 0.04) | 2.17(1.57,2.78) | 22(10 to 42) | 0.55(0.26 to 1.03) | 115(44 to 241) | 1.01(0.39 to 2.10) | 2.18(1.58,2.79) |
| **United States of America** | 298(118 to 539) | 0.44(0.17 to 0.79) | 48(21 to 84) | 0.06(0.02 to 0.10) | -6.22(-6.64,-5.80) | 13874(5488 to 25105) | 20.32(8.04 to 36.79) | 2237(955 to 3877) | 2.59(1.11 to 4.49) | -6.21(-6.60,-5.82) |
| **United States Virgin Islands** | 0(0 to 0) | 0.08(0.00 to 0.24) | 0(0 to 0) | 0.06(0.02 to 0.13) | -0.74(-1.58,0.11) | 1(0 to 4) | 3.90(0.09 to 11.45) | 1(0 to 1) | 3.03(1.00 to 6.26) | -0.63(-1.42,0.17) |
| **Uruguay** | 1(0 to 2) | 0.13(0.04 to 0.25) | 1(1 to 3) | 0.15(0.05 to 0.29) | 0.44(-0.35,1.24) | 47(16 to 91) | 6.18(2.07 to 11.96) | 67(24 to 130) | 7.05(2.56 to 13.72) | 0.46(-0.33,1.25) |
| **Uzbekistan** | 6(2 to 13) | 0.20(0.07 to 0.41) | 17(9 to 27) | 0.18(0.10 to 0.29) | -0.23(-0.96,0.50) | 326(110 to 676) | 9.52(3.20 to 19.83) | 826(432 to 1332) | 8.92(4.67 to 14.38) | -0.18(-0.87,0.52) |
| **Vanuatu** | 0(0 to 0) | 0.01(0.00 to 0.05) | 0(0 to 0) | 0.03(0.01 to 0.07) | 2.21(1.79,2.64) | 0(0 to 1) | 0.66(0.12 to 2.24) | 1(0 to 2) | 1.31(0.36 to 3.43) | 2.21(1.93,2.49) |
| **Venezuela (Bolivarian Republic of)** | 14(6 to 24) | 0.39(0.17 to 0.66) | 14(6 to 27) | 0.18(0.08 to 0.35) | -2.37(-3.19,-1.55) | 687(297 to 1165) | 18.63(8.09 to 31.58) | 680(303 to 1294) | 8.69(3.87 to 16.55) | -2.36(-3.21,-1.51) |
| **Viet Nam** | 3(1 to 7) | 0.03(0.01 to 0.07) | 49(18 to 100) | 0.17(0.06 to 0.35) | 5.72(5.46,5.98) | 163(60 to 355) | 1.43(0.53 to 3.11) | 2267(846 to 4677) | 7.85(2.93 to 16.19) | 5.71(5.49,5.92) |
| **Yemen** | 1(0 to 2) | 0.05(0.02 to 0.11) | 7(3 to 16) | 0.11(0.04 to 0.24) | 2.42(1.90,2.94) | 45(16 to 97) | 2.37(0.85 to 5.04) | 353(130 to 760) | 5.34(1.99 to 11.50) | 2.45(1.92,2.97) |
| **Zambia** | 0(0 to 1) | 0.03(0.01 to 0.06) | 2(1 to 5) | 0.06(0.02 to 0.14) | 2.23(1.96,2.50) | 17(7 to 32) | 1.41(0.63 to 2.72) | 96(30 to 241) | 2.74(0.86 to 6.82) | 2.24(1.98,2.50) |
| **Zimbabwe** | 1(0 to 1) | 0.03(0.01 to 0.07) | 2(1 to 4) | 0.05(0.02 to 0.12) | 1.46(0.37,2.56) | 25(10 to 50) | 1.63(0.67 to 3.29) | 82(34 to 176) | 2.54(1.04 to 5.47) | 1.48(0.37,2.59) |

**Notes:** The rates are reported per 100,000 people per years. Data in parentheses are 95% uncertainty intervals for cases and age-standardized rates of mortality and DALYs, and 95% confidence intervals for AAPCs. **Abbreviations:** DALYs, disability-adjusted life-years; ASMR, age-standardized mortality rate; ASDR, age-standardized DALYs rate; AAPC, average annual percent change; UI, uncertainty interval; CI, confidence interval.
